# Systems Biology for Drug Target Discovery in Acute Myeloid Leukemia

**DOI:** 10.3390/ijms25094618

**Published:** 2024-04-23

**Authors:** Svetlana Novikova, Tatiana Tolstova, Leonid Kurbatov, Tatiana Farafonova, Olga Tikhonova, Natalia Soloveva, Alexander Rusanov, Victor Zgoda

**Affiliations:** Institute of Biomedical Chemistry, Pogodinskaya 10, 119121 Moscow, Russia; novikova@ibmc.msk.ru (S.N.) tolstova@ibmc.msk.ru (T.T.); kurbatovl@ibmc.msk.ru (L.K.); farafonova.tatiana@ibmc.msk.ru (T.F.); ovt@ibmh.msk.su (O.T.); n.solovyova@ibmc.msk.ru (N.S.); rusanov@ibmc.msk.ru (A.R.)

**Keywords:** all-trans-retinoic acid, acute myeloid leukemia, HL-60, NB4, K562, granulocytic differentiation, proteome, transcriptome

## Abstract

Combining new therapeutics with all-*trans*-retinoic acid (ATRA) could improve the efficiency of acute myeloid leukemia (AML) treatment. Modeling the process of ATRA-induced differentiation based on the transcriptomic profile of leukemic cells resulted in the identification of key targets that can be used to increase the therapeutic effect of ATRA. The genome-scale transcriptome analysis revealed the early molecular response to the ATRA treatment of HL-60 cells. In this study, we performed the transcriptomic profiling of HL-60, NB4, and K562 cells exposed to ATRA for 3–72 h. After treatment with ATRA for 3, 12, 24, and 72 h, we found 222, 391, 359, and 1032 differentially expressed genes (DEGs) in HL-60 cells, as well as 641, 1037, 1011, and 1499 DEGs in NB4 cells. We also found 538 and 119 DEGs in K562 cells treated with ATRA for 24 h and 72 h, respectively. Based on experimental transcriptomic data, we performed hierarchical modeling and determined cyclin-dependent kinase 6 (CDK6), tumor necrosis factor alpha (TNF-alpha), and transcriptional repressor CUX1 as the key regulators of the molecular response to the ATRA treatment in HL-60, NB4, and K562 cell lines, respectively. Mapping the data of TMT-based mass-spectrometric profiling on the modeling schemes, we determined CDK6 expression at the proteome level and its down-regulation at the transcriptome and proteome levels in cells treated with ATRA for 72 h. The combination of therapy with a CDK6 inhibitor (palbociclib) and ATRA (tretinoin) could be an alternative approach for the treatment of acute myeloid leukemia (AML).

## 1. Introduction

Acute myeloid leukemia (AML) is a group of disorders of the myeloid lineage of hematopoiesis. French–American–British (FAB) and World Health Organization (WHO) classification systems distinguish various subtypes of AML based on the morphology and the expression profile of cluster of differentiation (CD) markers or on the basis of a genetic profile, respectively [1]. Both classification systems show the heterogeneity of the disease and each AML subtype requires specific treatment, which differs by its effectiveness. The acute promyelocytic leukemia (APL) subtype of AML is cured by chemotherapy-free regimens combining all-*trans*-retinoic acid (ATRA) and arsenic trioxide with cure rates of 90% [2] and stands out compared to other AML subtypes. In normal promyelocytic cells, ATRA, which is derived from vitamin A, binds RARA/RARX dimers and triggers cell maturation into granulocytes. In turn, APL cells carry a characteristic mutation, i.e., translocation between 15 and 17 chromosomes t(15;17), that affects RARalpha and PML genes, leading to chimeric PML/RARalpha protein expression and impairing its function. Damaged receptors provide high avidity from PML/RARalpha for the nuclear corepressor N-CoR/histone deacetylase (HDAC) complex that blocks granulocytic differentiation [3]. Since the high dosage of ATRA reverses the differentiation block, the ATRA-based regimens constitute targeted APL therapy. It is also important to emphasize that, unlike conventional chemotherapy, retinoids do not kill tumor cells but differentiate them into mature neutrophils. This could explain the high effectiveness of the treatment of APL with ATRA.

For a long time, the standard treatment for most other types of AML included anthracycline and cytarabine-based chemotherapy. The 5-year overall survival rate is about 30–40% in adults, decreasing down to 10–15% in older patients (age 60 years and older) [2]. The AML relapse rate is high, which explains the low treatment efficacy. Recent studies of the AML molecular landscape at the genome and transcriptome levels highlighted the clinical significance of mutation in numerous genes, e.g., *WT1*, *FLT3-ITD* (mutation in the tyrosine kinase domain), IDH1, IDH2, KMT2A, NPM1, NUP98-NSD1, *ASXL1*, *RUNX1*, *TP53,* and *DNMT3A* [4,5]. The heterogeneity of the mutational status among AML patients complicates the selection of the most appropriate treatment. Yet, the use of targeted therapy, depending on the patient’s mutational profile, has been gaining momentum. Nine new treatment protocols have been approved by the FDA since 2017 [2].

The cell lines HL-60 and NB4 have been popular for decades as the model objects for studying the biology of leukemia. The NB4 cells, representing APL subtypes of leukemia, carry the characteristic t(15;17) mutation, while HL-60 cells, recognized as AML subtypes, have deletions in the *TP53* gene as well as amplification of the *c-MYC* gene. The *TP53* mutation accounts for approximately 5–20% of AML cases. It is associated with very poor survival outcomes after standard therapeutic treatments. In the context of increasingly popular targeted therapy for AML, one approach to treating *TP53*-mutated tumors is to reactivate the tumor suppressor by eprenetapopt (APR-246) or magrolimab [6]. However, HL-60 cells as well as NB4 cells respond to ATRA treatment, resulting in their differentiation into neutrophils [7]. There are attempts to introduce ATRA-based treatment to non-APL subtypes of AML, e.g., the combination of GSK3 inhibition and tretinoin [8].

Molecular events initiated in cells exposed to ATRA and involving changes in thousands of RNAs and proteins may be detected during transcriptome and proteome profiling performed by means of modern omics technologies. These molecules, which could be crucial for granulocyte differentiation, represent potential drug targets. The appropriate interpretation of omics data is important for elucidating the molecular mechanisms underlying granulocyte maturation. Direct annotations using open databases and canonical pathways (e.g., GO, Reactome, KEGG) represent a powerful tool to interpret omics data. Using GO and KEGG database annotations for the analysis of the SILAC-based proteome profiling of HL-60 cells treated with HDAC inhibitors, the differentially expressed proteins (DEPs) involved in cell division (P27, cyclinE, Cdk2, components of SWI/SNF-related complexes) have been elucidated [9]. It was also shown that these DEPs represented the parts of the mechanism of decreased proliferation under HDAC inhibitor treatment.

At the same time, the direct annotation of differentially expressed molecules is limited in the discovery of fundamentally new regulatory molecules. In addition, upstream regulatory molecules, such as transcription factors (TFs), cell surface receptors, and regulatory kinases, may be responsible for the experimentally observed quantitative changes in transcripts and proteins. In a recent study, the transcriptome profiling of ATRA-treated NB4 cells followed by upstream regulator search resulted in the identification of CEBPε, IRF1, PU.1, and STAT1 proteins that could regulate the molecular response to ATRA [10]. In our previous work, we performed a time-course study of the ATRA effects on the transcriptome and proteome of HL-60 cells. Using upstream regulator search, we found poly (ADP-ribose) polymerase (PARP1) and TF HIC1 as regulators of ATRA-induced granulocytic differentiation [11].

In the current work, we have applied an integrative bioinformatic approach to build modeling schemes of molecular changes induced in NB4, HL-60, and K562 cells by ATRA treatment.

## 2. Results

### 2.1. Transcriptomic Analysis Revealed Common DEGs Involved in the Immune Response in ATRA-Responsive HL-60 and NB4 Cells

Genome-scale transcriptome analysis is a useful tool for identification of the molecular changes in the early steps of ATRA-induced leukemic cell differentiation. We have studied the transcriptome response in two ATRA-responsive cell lines: HL-60 and NB4. As evidenced by the morphology and expression of convenient granulocyte markers (CD11b and CD38), these cells acquired the phenotype of mature granulocytes (Figure S1) after the ATRA treatment for 72 h.

**Figure 1:**
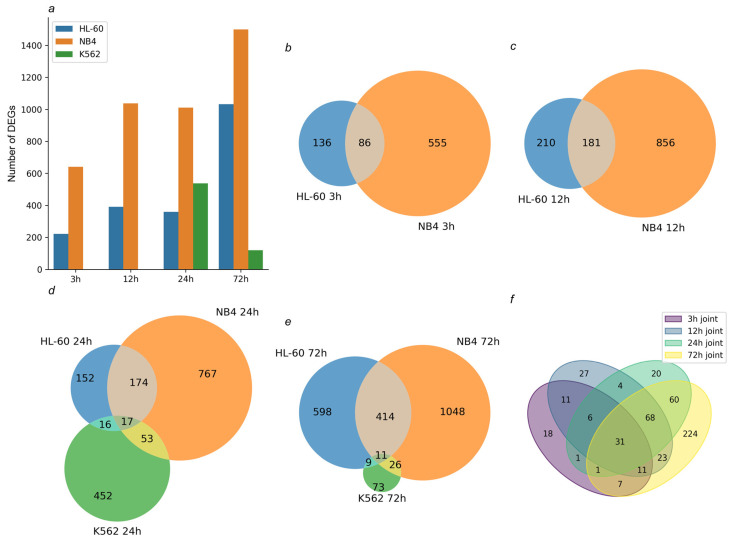
Transcriptomic profiling of the studied cells after the ATRA treatment. (**a**) Bar chart demonstrates the number of differentially expressed genes (DEGs) (*p*-value < 0.05, fold change (FC) > 2) identified in HL-60 and NB4 cells treated with ATRA for 3 h, 12 h, 24 h, and 72 h and in K562 cells treated with ATRA for 24 h and 72 h. (**b**,**c**) Venn diagrams show overlap of DEGs identified in the ATRA-responsive HL-60 and NB4 cells exposed to ATRA for 3 h and 12 h, respectively. (**d**,**e**) Venn diagrams show overlap of DEGs identified in HL-60, NB4, and K562 cells exposed to ATRA for 24 h and 72 h, respectively. (**f**) Venn diagram shows overlap of DEGs common for ATRA-responsive HL-60 and NB4 cells exposed to ATRA for 3 h, 12 h, 24 h, and 72 h.

We have also studied the transcriptome profiles of K562 cells, which did not express CD11b and CD38 granulocyte markers under the ATRA treatment (Figure S1). The transcriptomic profiling of HL-60 and NB4 cells was performed after their treatment with ATRA for 3 h, 12 h, 24 h, and 72 h. The transcriptomic profiling of K562 cells was performed after the ATRA treatment for 24 h and 72 h. A total of 17,346, 17,683, and 16,342 gene expressions were detected in HL-60, NB4, and K562 cells, respectively, at all the time points studied. The data were uploaded to BioStudies and are available with the accession number E-MTAB-13723. Differentially expressed genes (DEGs) (*p*-value < 0.05, fold change (FC) > 2) were determined for each cell line studied (Table S1, Figure 1).

**Figure 2:**
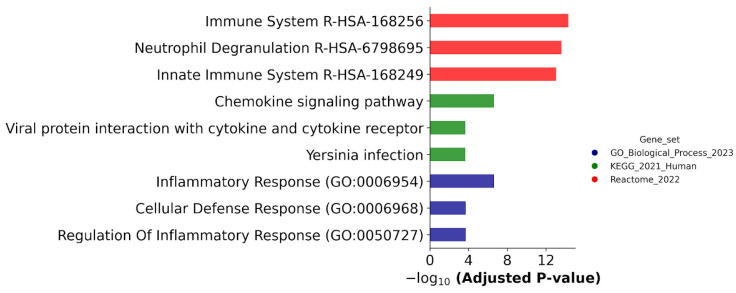
Biological annotation of DEGs, which were detected in both ATRA-responsive HL-60 and -NB4 cells exposed to the ATRA treatment. The DEGs that were revealed at 3, 12, 24, and 72 h after the ATRA treatment, and common for cell lines HL-60 and NB4, were combined. The DEGs were annotated against the GeneOntology (Biological process), KEGG, and Reactome databases. The top three most significant groups are shown. The significance of the adjusted *p*-value cut-off was <0.05 (gseapy library v. 1.0.4).

The genome-scale transcriptomic analysis identified 222, 391, 359, and 1032 DEGs in ATRA-responsive HL-60 cells, treated with ATRA for 3 h, 12 h, 24 h, and 72 h, respectively (*p*-value < 0.05). Also, 641, 1037, 1011 and 1499 DEGs were detected in ATRA-responsive NB4 cells treated with ATRA for 3 h, 12 h, 24 h, and 72 h, respectively (*p*-value < 0.05). These included 86, 181, 191, and 425 DEGs common for the ATRA-responsive HL-60 and NB4 cells (Figure 1b–f). The functional annotation of this group of DEGs is presented in Figure 2 and Table S1.

Figure 2 shows that the DEGs common for both ATRA-responsive cells treated with ATRA were assigned to the Inflammatory Response (GO:0006954), Dendritic Cell Migration (GO:0036336), and Regulation of Inflammatory Response (GO:0050727) groups according to the GeneOntology (GO) database (Biological process); common DEGs belonged to the Chemokine signaling pathway, Viral protein interaction with cytokine and the cytokine receptor pathway, and Yersinia infection pathway according to the KEGG database; and common DEGs were involved in the Immune System R-HSA-168256, Neutrophil Degranulation R-HSA-6798695, and Innate Immune System R-HSA-168249 processes according to the Reactome database.

### 2.2. DEGs Common for All Cells Exposed to the ATRA Treatment Were Enriched with Cell-Death-Regulating Components

The K562 cells do not express the granulocytic differentiation markers and do not change phenotype in response to the ATRA treatment. However, after exposure to ATRA for 24 and 72 h, we found 538 and 119 DEGs in these cells. In contrast to the ATRA-responsive cells, in which the number of DEGs increased up to 72 h, the transcriptomic response of K562 cells to ATRA had already decreased by this time. Moreover, 17 DEGs were overlapped in all three cell lines studied at 24 h after the ATRA treatment (Figure 1d and Figure S2), and 16 DEGs were common for the ATRA-responsive HL-60 cells and ATRA-non-responsive K562 cells (Figure 1d and Figure S3). Most of these DEGs were up-regulated in HL-60 cells but down-regulated in K562 cells (Figure S3). A larger intersection of 52 DEGs was observed between K562 and NB4 cells (Figure 1d and Figure S4). Down-regulated genes showed congruent profiles in both cell lines (Figure S4). Also, 11, 9, and 26 DEGs were overlapped between the three studied lines—between HL-60, NB4, and K562 cells, between K562 and HL-60 cells and between K562 and NB4 cells—at the 72 h time point of the ATRA treatment (Figure 1e and Figures S5–S7).

**Figure 3:**
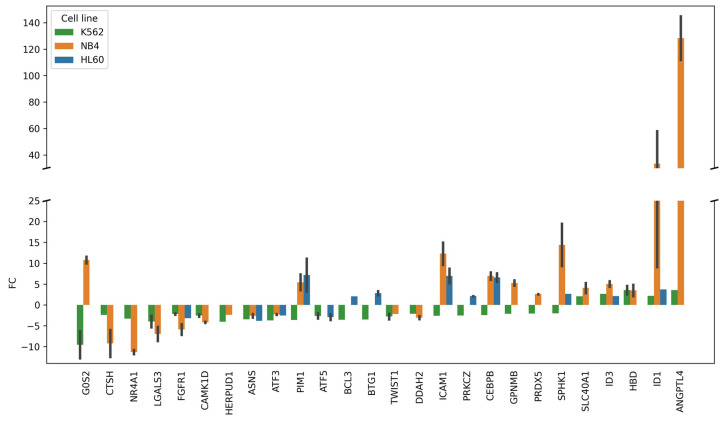
The transcriptomic profile of DEGs belonged to the group of “Regulation of cell death” (GO:0010941) according to the GO annotation (“Biological process” category). The group included 26 DEGs common for ATRA-non-responsive K562 cells and ATRA-responsive NB4 cells and/or HL-60 at the 24 h and/or 72 h time points of the ATRA treatment as compared to control (0 h). FC—fold change (adjusted *p*-value < 0.05). Error bars are shown for DEGs, for which fold change (FC) was determined at both the 24 and 72 h time points of the ATRA treatment.

The biological annotation against GO terms (Biological processes) shows that DEGs common for the three cell lines studied were enriched with the components (26 DEGs) of the “Regulation of cell death” (GO:0010941) group (Table S1). The profile of these DEGs is shown in Figure 3.

Figure 3 demonstrates that 10 DEGs (*G0S2*, *PIM1*, *BCL3*, *BTG1*, *ICAM1*, *PRKCZ*, *CEBPB*, *GPNMB*, *PRDX5*, and *SPHK1*) were up-regulated in ATRA-responsive HL-60 and/or NB4 cells and, at the same time, down-regulated in ATRA-non-responsive K562 cells. Among the DEGs regulating cell death, the most prominent changes were observed for up-regulated ANGPTL4 (FC = 144 in NB4 cells).

**Figure 4:**
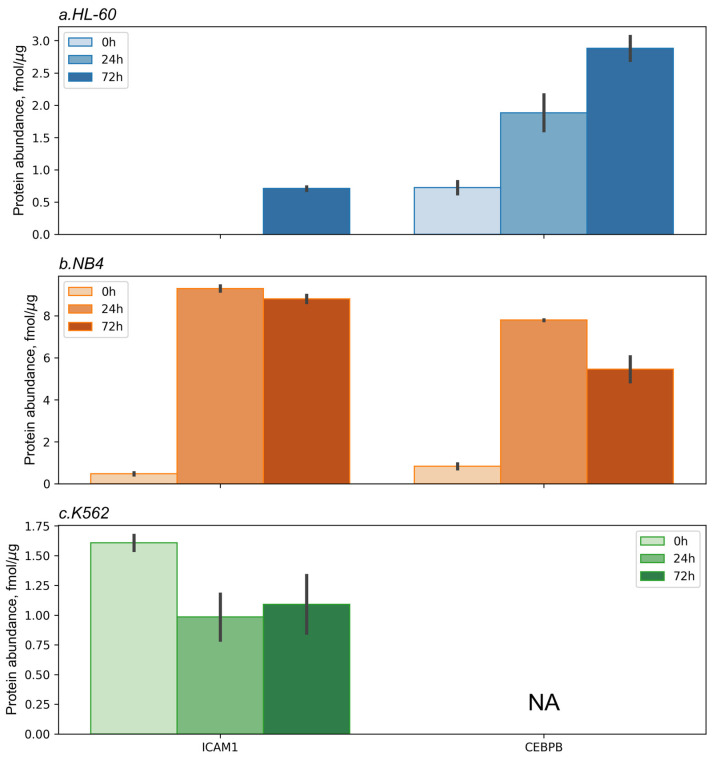
Protein expression levels of ICAM1 and CEBPB measured by targeted mass spectrometry in the selected reaction monitoring (SRM) mode using isotope-labeled standard (SIS) peptides (SRM/SIS) in HL60 (**a**), NB4 (**b**), and K562 (**c**) cell lines at 0 h, 24 h, and 72 h after the ATRA treatment. We performed SRM/SIS analysis in two biological replicates. The *y*-axis shows the protein abundance in fmol/µg of total protein.

For all the cell lines studied, we performed targeted mass spectrometric analysis of ICAM1 (CD54) and CEBPB proteins at 0 h, 24 h, and 72 h after the ATRA treatment (Figure 4 and Figure S8). SRM/SIS analysis showed a significant (*p*-value < 0.05) increase in CEBPB protein content up to 3.8-fold and 6.8-fold in HL-60 and NB4 cells, respectively, at 72 h after the ATRA addition. The peptide that was mapped to CEBPB protein was not detected in the K562 cell line. The expression of ICAM1 (CD54) protein was found to be increased (18.5-fold at 72 h after the ATRA addition) in the NB4 cell line at 72 h after the ATRA treatment. Also, ICAM1 (CD54) protein was detected in HL-60 cells at the levels of 0.7 ± 0.4 fmol/µg of total protein at 72 h after the ATRA treatment. In the K562 cell line, SRM/SIS analysis showed a decrease (1.7-fold) in content of ICAM1 (CD54) protein at 24 h after the ATRA addition.

### 2.3. The Most Up- and Down-Regulated DEGs in the ATRA-Treated Cells Are Associated with Metabolism of Retinoids

**Figure 5:**
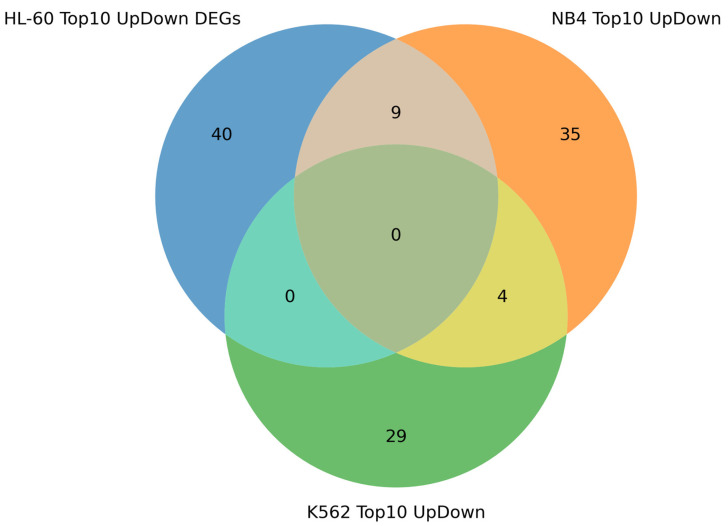
Venn diagram showing intersection of top 10 most up- and down-regulated DEGs found in HL-60, NB4, and K562 cells exposed to ATRA. The top 10 most up- and down-regulated DEGs found at the 3 h, 12 h, 24 h, and 72 h time points of the ATRA treatment were combined within each cell line studied.

Considering that the most prominent alteration in gene expression could be associated with regulatory function, we have determined the top 10 up- and down-regulated DEGs (adjusted *p*-value < 0.05) chosen by degree of fold change at the 3 h, 12 h, 24 h, and 72 h time points of the ATRA treatment for each cell line studied (Figures S9–S11). The result of the analysis is presented in Figure 5.

Figure 5 demonstrates that nine DEGs were common for ATRA-responsive cell lines (HL-60 and NB4). The genes, *CCL2*, *ID1*, *MYBPH*, *NCF1*, *DHRS3*, and *PALLD,* were the most up-regulated DEGs in HL-60 and NB4 cells, and *GPR68, MICAL2*, and *VIM* were the most down-regulated DEGs in HL-60 and NB4 cells. The *DHRS3* gene product catalyzes NADPH-dependent reduction of all-*trans*-retinal to all-*trans*-retinol.

*ANGPTL4* and *CYP26A1* were among the most up-regulated genes in both ATRA-responsive NB4 cells and in ATRA-non-responsive K562 cells, which do not acquire the neutrophil phenotype. In NB4 cells, *ANGPTL4* was 112-fold- and 145-fold-up-regulated at the 24 h and 72 h time points of the ATRA treatment, respectively. In K562 cells, it was 3.5-fold-up-regulated at the 72 h time point of the ATRA treatment. In NB4 cells, *CYP26A1* was 162-fold- and 840-fold-up-regulated at the 24 h and 72 h time points of the ATRA treatment, respectively. Notably, in K562 cells, the level of *CYP26A1* encoding the retinoid metabolizing cytochrome [12,13] was 28-fold-and 24-fold-up-regulated at the 24 h and 72 h time points of the ATRA treatment (Figure S11).

### 2.4. Modeling of the Molecular Response in HL-60, NB4, and K562 Cells Exposed to the ATRA Treatment Revealed Cyclin-Dependent Kinase 6 (CDK6), Tumor Necrosis Factor Alpha (TNF-Alpha), and Transcriptional Repressor CUX1 as the Key Regulators

**Figure 6:**
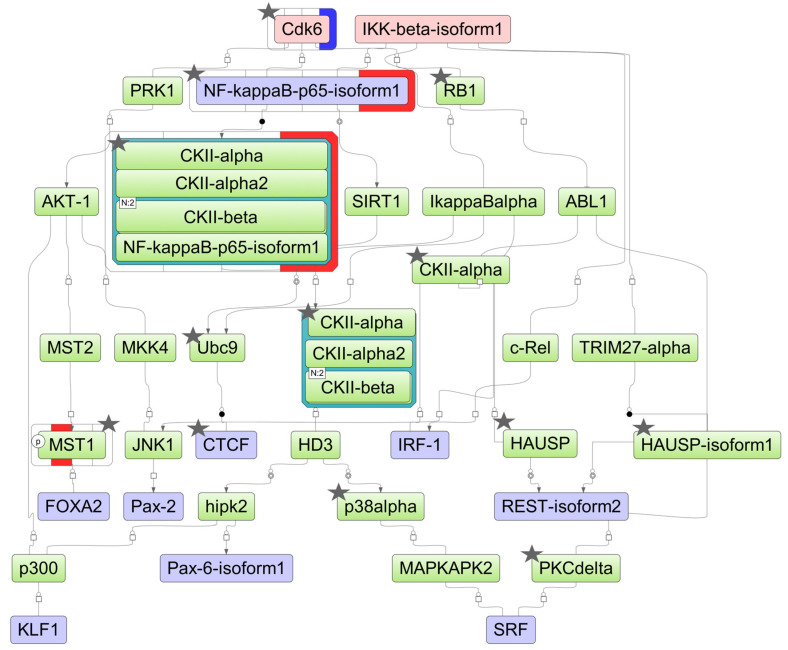
The model network of HL-60 cells underlying the response to ATRA treatment based on transcriptomic data. Key regulatory molecules are denoted by pink ellipses; the connecting molecules considered by the graph-analyzing algorithm to find the path from the TF input list to the key molecule are denoted by green ellipses; the TF molecules are denoted by lilac ellipses. The colored bars around molecules show changes in the decreased (blue) and increased (red) protein expression at the 3 h, 12 h, 24 h, and 72 h time points of the ATRA treatment. Molecules identified at the proteome level by at least two unique peptides are marked by asterisks.

**Figure 7:**
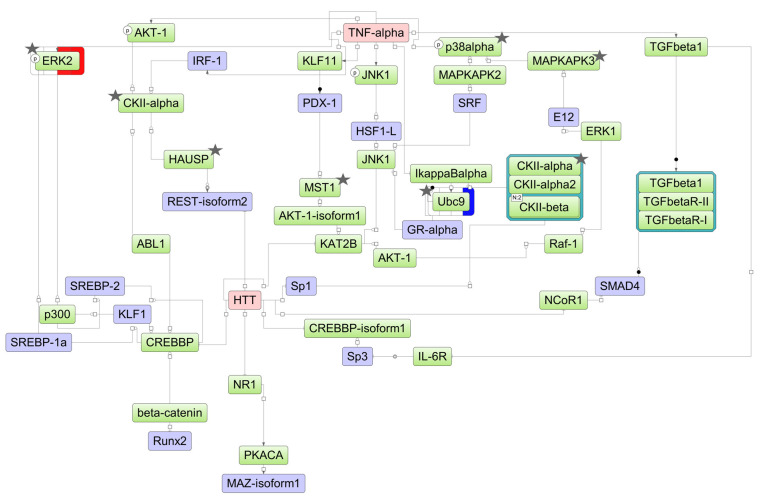
The model network of NB4 cells underlying the response to ATRA treatment based on transcriptomic data. Key regulatory molecules are denoted by pink ellipses; the connecting molecules considered by the graph-analyzing algorithm to find the path from the TF input list to the key molecule are denoted by green ellipses; the TF molecules are denoted by lilac ellipses. The colored bars around molecules show changes in the decreased (blue) and increased (red) protein expression at the 3 h, 12 h, 24 h, and 72 h time points of the ATRA treatment. Molecules identified at the proteome level by at least two unique peptides are marked by asterisks.

**Figure 8:**
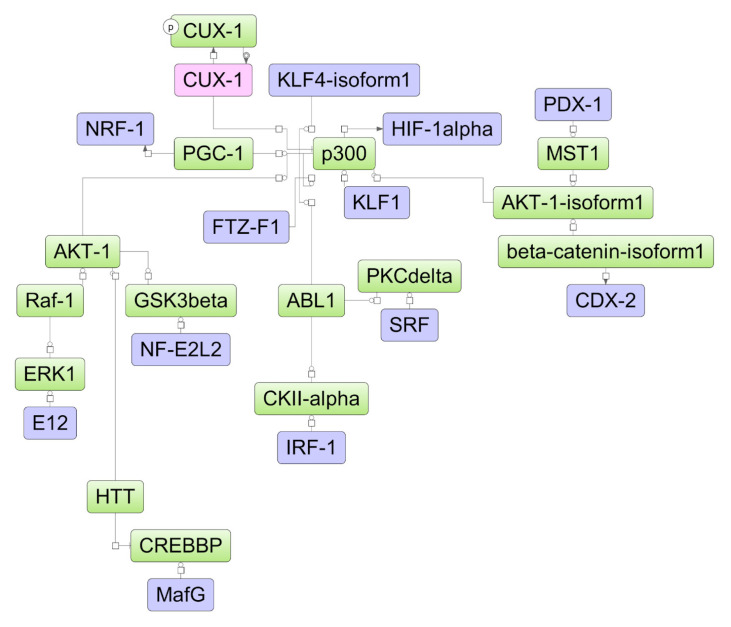
The model network of K562 cells underlying the response to ATRA treatment based on transcriptomic data. Key regulatory molecules are denoted by pink ellipses; the connecting molecules considered by the graph-analyzing algorithm to find the path from the TF input list to the key molecule are denoted by green ellipses; the TF molecules are denoted by lilac ellipses.

The DEGs found in the experiment may be affected by certain transcription factors (TFs), which, in turn, may be influenced by upstream regulators in the pathways. Assuming such a hierarchical regulatory system, we have developed a model scheme of intermolecular interactions for each cell line studied by using the TRANSPATH^®^ database incorporated in the geneXplain platform (Figure 6, Figure 7 and Figure 8). Using this approach, we determined 25, 35, and 37 TFs potentially regulating DEGs in HL-60, NB4, and K562 cells, respectively (Table S2). These included nine TFs (IRF1, SRF, MAZ, MEIS1, PATZ1, KLF1, INSM1, MZF1, and EBF1) common for all cell lines studied, while the REST and ZBTB33 TFs were identified as regulatory TFs for both ATRA-responsive lines HL-60 and NB4. Using the TRANSPATH^®^ database, the geneXplain platform software (version 7.3) predicted potential key molecules and visualized model pathways triggered in response to ATRA treatment in the ATRA-responsive cell lines HL-60 and NB4, as well as in the ATRA-non-responsive K562 cell line (Figure 6, Figure 7 and Figure 8).

For HL-60 cells, the key molecules of the model pathway included cyclin-dependent kinase 6 (CDK6) and inhibitor of nuclear factor kappa B kinase subunit beta (IKK-beta) (Figure 6). The key molecule CDK6 was identified at the proteomic level, with both transcript and protein levels significantly reduced at the 72 h time point of the ATRA treatment. The molecules p38alpha (MAPK14), CTCF, RB1, CDK6, CKIIalpha (CSNK1A1), CKIIbeta (CSNK2B), UBC9 (UBE2I), PKCdelta (PRKCD), HAUSP (USP7), nuclear factor NF-kappa-B p65 subunit (RELA), and MST1 (STK4) were identified at the proteome level by at least two unique peptides (Table S2).

For NB4 cells, the key molecule of the model scheme was tumor necrosis factor-alpha (TNF-alpha) (Figure 7), a pro-inflammatory cytokine involved in regulation of the proliferation/differentiation balance. TNF-alpha transcript abundance decreased significantly starting at the 24 h time point of the ATRA treatment. The molecules p38alpha (MAPK14), CKIIalpha (CSNK1A1), CKIIbeta (CSNK2B), UBC9 (UBE2I), HAUSP (USP7), ERK2 (MAPK1), MAPKAPK3, and MST1 (STK4) were identified at the proteome level by at least two unique peptides (Table S2).

The transcriptional repressor *CUX1* was identified as a key molecule potentially regulating the molecular response to ATRA in K562 cells (Figure 8). There were no proteins identified in the mass-spectrometry experiment. The differential expression for elements of the model scheme was not determined at the transcriptome proteome levels of K562 cells.

### 2.5. Proteomics of HL-60, NB4, and K562 Cells Exposed to the ATRA Treatment

Potentially, the elements of model pathways, especially key molecules, expressed at the proteome level and demonstrating quantitative changes are of particular interest as pharmacological targets that potentiate the differentiating effect of ATRA.

**Figure 9:**
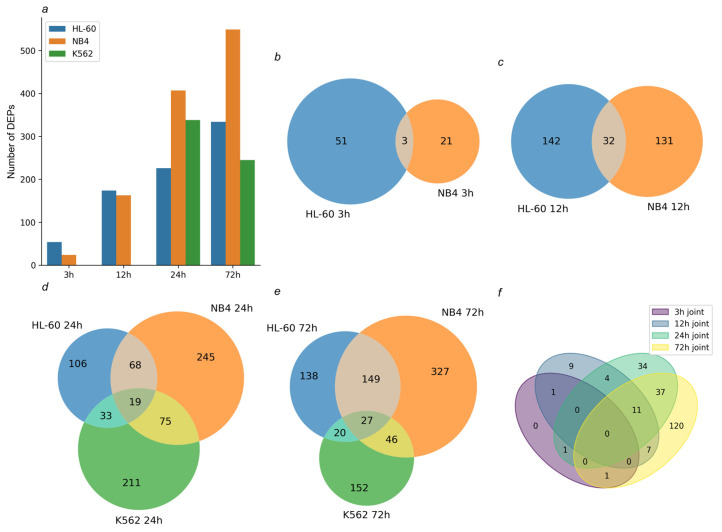
Tandem mass tag-based quantitative proteomic profiling of cells treated with ATRA. (**a**) Bar chart demonstrates the number of differentially expressed proteins (DEPs) identified in HL-60, NB4, and K562 cells at the 3 h, 12 h, 24 h, and 72 h time points of the ATRA treatment. (**b**,**c**) Venn diagrams show overlap of DEPs identified in ATRA-responsive HL-60 and NB4 cells at the 3 h and 12 h time points of the ATRA treatment, respectively. (**d**,**e**) Venn diagrams show overlap of DEPs identified in HL-60, NB4, and K562 cells at the 24 h and 72 h time points of the ATRA treatment, respectively. (**f**) Venn diagram shows overlap of DEPs common for ATRA-responsive cell lines HL-60 and NB4 at 3 h, 12 h, 24 h, and 72 h time points of the ATRA treatment.

For the elucidation of differentially expressed protein (DEPs) in HL-60, NB4, and K562 cells exposed to the ATRA treatment, we performed proteomic profiling using semi-quantitative mass-spectrometry with tandem tags (Table S3, Figure 9). The mass spectrometry proteomics data have been deposited in the ProteomeXchange Consortium via the PRIDE partner repository with the dataset identifier PXD050317. We identified 1576, 1590, and 1682 proteins by at least two unique proteotypic peptides (FDR < 0.01) in HL-60, NB4, and K562 cells, respectively (Table S3). These included 1155 proteins identified by at least two unique proteotypic peptides (FDR < 0.01) in all three cell lines studied (Figure S12).

In HL-60 cells, 54, 174, 226, and 334 DEPs were determined at the 3 h, 12 h, 24 h, and 72 h time points of the ATRA treatment, respectively. In NB4 cells, 24, 163, 407, and 549 DEPs were determined at the 3 h, 12 h, 24 h, and 72 h time points of the ATRA treatment, respectively. In K562 cells, 338 and 245 DEPs were determined at the 24 h and 72 h time points of the ATRA treatment, respectively.

**Figure 10:**
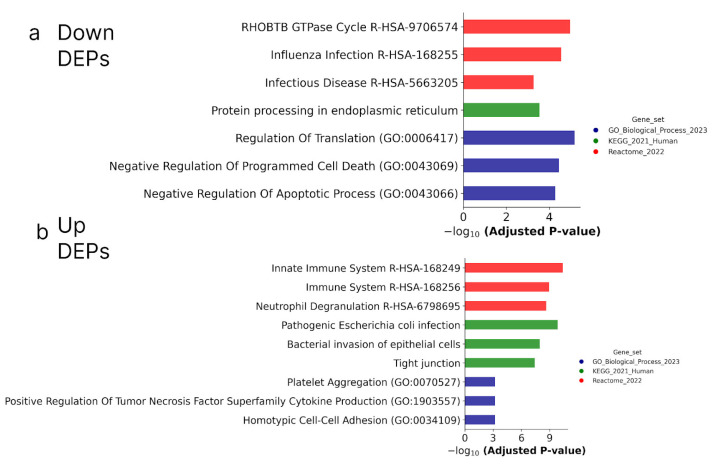
Biological annotation of up-regulated (**a**) and down-regulated (**b**) DEPs in both ATRA-responsive HL-60 and NB4 cells treated with ATRA. The DEPs detected at the 3 h, 12 h, 24 h, and 72 h time points of the ATRA treatment, and common for HL-60 and NB4 cells, were combined. The DEPs were annotated against GeneOntology (Biological process), KEGG, and Reactome databases. The top three most significant groups are shown. The significance of the adjusted *p*-value cut-off was <0.05 (gseapy library v. 1.0.4).

**Figure 11:**
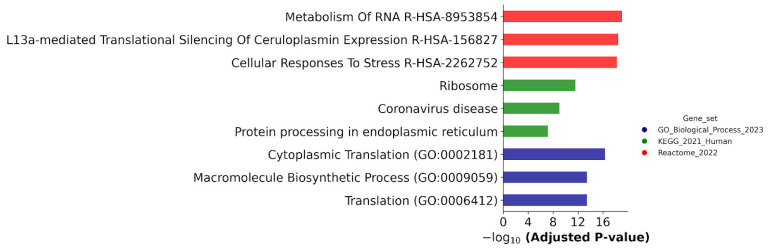
Biological annotation of DEPs determined in ATRA-non-responsive K562 cells and ATRA-responsive NB4 and/or HL-60 cells at the 24 h and/or 72 h time points of the ATRA treatment as compared to control (0 h). The DEPs were annotated against GeneOntology (Biological process), KEGG, and Reactome databases. The top three most significant groups are shown. The significance of the adjusted *p*-value cut-off was <0.05 (gseapy library v. 1.0.4).

To evaluate the biological significance of the resulting proteins, we performed functional annotation in the GeneOntology (GO, Biological processes, cellular component, cellular localization), Kyoto Encyclopedia of Genes and Genomes (KEGG, Biological processes), and Reactome Pathways (Biological processes) databases (Figure 10 and Figure 11).

Figure 10 shows that according to biological annotation, the proteins up-regulated during the ATRA treatment of the ATRA-responsive HL-60 and NB4 cells were mostly involved in the function of the immune system (as it was observed for transcriptomic data). At the same time, biological annotation against the GeneOntology (GO) database (Biological process) revealed an enrichment of down-regulated DEPs with proteins involved in the regulation of translation and negative regulation of apoptosis (NPM1, TFRC, PA2G4, ATAD3A, HSP90B1, HSPD1, DNAJA1, PRDX2, UFM1, FLNA, NAA15, PTMA, and TXNDC5) (Table S3).

Figure 11 demonstrates that biological annotation against the Reactome database revealed an enrichment of DEPs with proteins involved in the metabolism of RNA and cellular responses to stress; they were found in all cell lines studied.

In both ATRA-responsive HL-60 and NB4 cells, *AHNAK*, *CA2*, *ESYT1*, *PRG2*, *SRM,* and *VIM* were down-regulated at the transcript and protein levels, while *ALOX5AP*, *ARPC1B*, *CD38*, *CTSD*, *CYBB, ITGB2*, *MYO1F*, *NCF1*, *PGD*, *PLEK*, *POR*, *PRAM1*, *PTPN6*, *SAMHD1*, *SERPINB1*, *SH3BGRL3*, *SLC16A3*, and *WIPF1* were up-regulated in these cells at both transcript and protein levels.

Changes in the molecular landscape in response to ATRA treatment were more prominent for the NB4 cells than for the HL-60 cells at the transcriptome and proteome levels. For both ATRA-responsive cell lines (HL-60 and NB4), the number of up-regulated DEGs with increased expression (DEGup) at the early time points (3 h and 12 h) of the ATRA treatment significantly exceeded (2-fold) the number of down-regulated DEGs (DEGdown); however, at the 72 h time point, the number of DEGup and DEGdown was almost equal (Figure S13). This observation may indicate that, at the transcriptome level, induced granulocytic differentiation begins primarily through gene activation, gradually reaching the activation/suppression balance. At the proteome level, the number of DEPdown and DEPup was similar at the early time points (3 h and 12 h) of the ATRA treatment, and then the number of DEPdown increased steadily as granulocytic differentiation progressed for both ATRA-responsive lines, and after 24 h of incubation with ATRA, the number of DEPup exceeded, which in turn reached a plateau (Figure S13).

## 3. Discussion

High-throughput omics experiments allow for characterization of the molecular responses in leukemic cells under the ATRA treatment. Time-course transcriptomic and proteomic analysis demonstrated a pronounced molecular response not only in ATRA-responsive HL-60 and NB4 cell lines, but also in ATRA-non-responsive cells K562 cells treated with retinoid. Straightforward annotations using open databases and canonical pathways (e.g., GO, Reactome, KEGG) showed that differentially expressed genes (DEGs) and proteins (DEPs) were enriched with immune system components, retinoid metabolic enzymes, regulators of apoptosis, and components of the endoplasmic reticulum stress response. Finally, applying upstream regulator search, we determined cyclin-dependent kinase 6 (CDK6), tumor necrosis factor alpha (TNF-alpha), and transcriptional repressor CUX1 as the key regulators that potentially trigger a molecular response to the ATRA treatment in HL-60, NB4, and K562 cell lines, respectively.

As expected, the DEGs overlapped between HL-60 and NB4 cells exposed to the ATRA treatment. In these ATRA-responsive cells, the flow cytometry analysis revealed the expression of the granulocyte markers (CD11b and CD38) after the ATRA treatment. The involvement of HL-60 and NB4 DEGs in the functioning of the immune system, including neutrophils, reflected the acquisition of the granulocytic phenotype by leukemia cells. However, we could not reveal a particular regulatory mechanism of ATRA-induced granulocytic differentiation based on DEGs in ATRA-responsive cells alone.

In addition to the cell lines HL-60 and NB4 that are convenient cell models to study granulocytic differentiation [7], we assessed the molecular response to the ATRA treatment in the K562 cells that represent the culture model of chronic myelogenous leukemia (CML). In clinical studies, ATRA was not effective in treating CML patients [14]. However, these cells are used to study the ATRA effects in combination with other therapeutic approaches such as the inhibition of sphingosine kinases (SphKs) [15]. Since, in our experiments, K562 cells exposed to ATRA did not express the CD11b and CD38 granulocytic markers, they were defined as ATRA-non-responsive cells. At the same time, there are DEGs that overlap between the transcriptomic data obtained for ATRA-responsive and -non-responsive cell lines. Interestingly, the transcriptome response of NB4 and K562 cells was more similar as compared to HL-60 and K562 cell transcriptome changes.

The DEGs which overlapped between all three cell lines exposed to ATRA were enriched in components regulating apoptosis. Some of them (*G0S2*, *PIM1*, *BCL3*, *BTG1*, *ICAM1* (*CD54*), *PRKCZ*, *CEBPB*, *GPNMB*, *PRDX5*, and *SPHK1*) demonstrated a non-congruent differential expression profile (up-regulation in the ATRA-responsive HL-60 and NB4 cells and down-regulation in ATRA-non-responsive K562 cells). In addition to the regulation of apoptosis, the protein products of these genes are involved in signaling pathways that are associated with cancer development and progression, including NF-ĸB (*BCL3, SPHK1*, and *PRKCZ*) and c-MYC (*PIM1* and *BTG1*). The protein product of the *BTG1* gene possesses antiproliferative features regulating gene expression at the transcriptional and post-transcriptional levels [16]. Taking into consideration genetic mutations detected in HL-60 cells (e.g., *c-MYC* amplification and *p53* deletion), *BTG1* could be suppressed by proto-oncogene *c-MYC* and, at the same time, could be activated in a p53-independent manner [16]. Moreover, *BTG1* is a target gene for histone deacetylase (HDAC) inhibitor chidamide [17].

Sphingosine kinase 1 (*SPHK1*) is another component of the group that included DEGs associated with the regulation of apoptosis. *SPHK1* was down-regulated in K562 cells but up-regulated in both ATRA-responsive cells, most prominently in NB4 cells (19-fold). It is characterized by complex and contradictory effects on cell survival [18]. Although *SPHK1* exhibits a proapoptotic effect, much attention has been focused on the relationship between high expression and tumor development, and, as a consequence, on the potential pharmacological *SPHK1* inhibition [19]. As was mentioned above, the inhibition of *SPHK1* in combination with ATRA treatment could be a therapeutic approach to CML treatment [15].

In the case of ATRA-responsive NB4 cells and ATRA-non-responsive K562 cells, *ANGPTL4* and *CYP26A1* were the most up-regulated DEGs induced by the ATRA treatment. In mammals, ATRA regulates various developmental processes, including brain formation and myeloid hematopoiesis [20]. At the same time, the optimal level of ATRA should be maintained during development, as retinoids are teratogenic [21]. The highly inducible enzyme of cytochrome P450 family 26 (CYP26), *CYP26A1*, is the key regulator of ATRA catabolism in mammals’ embryos and a predominant CYP26 enzyme in the adult human liver [12,13]. At the transcriptome level, another member of the CYP26 family, *CYP26B1*, was found to be up-regulated in our experiments in both NB4 (up to 119-fold) and K562 (up to 119-fold) cells. At the same time, no altered expressions of *CYP26A1* and *CYP26B1* were found in HL-60 cells treated with ATRA treatment. The inhibitors of CYP26 enzymes, e.g., talarozole, could be used to maintain the high ATRA levels and to prevent therapy resistance [13,22].

Angiopoietin-related protein 4 (*ANGPTL4*) was strongly up-regulated in NB4 (144-fold) cells at the transcriptome level. It is involved in migration and invasion and inhibited apoptosis in the case of colorectal cancer [23]. *ANGPTL4* is known to promote gefitinib resistance in lung cancer by regulating the NLRP3/ASC/Caspase 8 pathway [24]. In mouse AML models, an impairment of paired immunoglobulin-like receptor (PIRB), which binds proteins from the Angptls family, resulted in an increased differentiation of leukemia cells [25]. It is possible that *ANGPTL4* could be involved in molecular mechanisms of ATRA resistance.

Transcriptomic and proteomic methods separately provide valuable data. Using the transcriptomic analysis, it possible to register the early stages of response to pharmacological effects, while the data on protein expression are closer to the phenotypic features of cells and are closely related to the biological functions performed.

According to biological annotation, the DEPs up-regulated in both ATRA-responsive cell lines (HL-60 and NB4) were assigned to the various groups associated with immune system function, including neutrophil functions. The group of DEPs involved in neutrophil degranulation could be a new marker to assess granulocytic differentiation. These proteins could be added to the SRM method for analysis of the expression of markers CD33, CD97, CD54, CD38, CD18, CD11b, CD44, and CD71 during granulocytic differentiation that we developed recently [26]. The molecular features of AML may be employed in the search for prognostic biomarkers that would help in stratifying patients according to relapse-risk level, and to prescribe them a suitable treatment.

The molecules ARPC1B, CTSD, CYBB, ITGB2, NCF1, PTPN6, SAMHD1, SERPINB1, and WIPF1 that were annotated to be involved in the function of the immune system (DB Reactome, category Immune System R-HSA-168256) were up-regulated both at the transcriptome and proteome levels. According to the ProteinAtlas database, neutrophil cytosol factor 1 (NCF1), integrin beta-2 (ITGB2/CD18), tyrosine-protein phosphatase non-receptor type 6 (PTPN6), and WAS/WASL-interacting protein family member 1 (WIPF1) are mainly expressed in lymphoid tissue, bone marrow, spleen, and myeloid blood cells. On the other hand, a high expression of PTPN6 and SAMHD1 [27,28] correlates with poor prognosis for AML patients. These molecules could play a role in the mechanism of resistance to retinoid-based therapy.

Some of the DEPs down-regulated in both ATRA-responsive cell lines (HL-60 and NB4) and assigned to the Negative Regulation of Programmed Cell Death (GO:0043069) group are considered the targets for therapeutics used in the treatment of solid cancers. Kinase inhibitor sorafenib impairs prothymosin alpha PTMA expression in hepatocellular carcinoma (HCC) [29]. Ainsliadimer A, a sesquiterpene lactone dimer with antitumor activity, inhibits PRDX2 and shows an antitumor effect in colorectal cancer cells [30]. The inhibition of DNAJA1 increases the sensitivity to radiation or chemotherapy treatment in cancer cells carrying mutated p53 [31]. Targeting these molecules could also enhance the antileukemic effect of ATRA.

Furthermore, the DEPs found in all cell lines studied are associated with cellular responses to stress, which could be of a genotoxic nature. Enhanced cell survival leads to high levels of DNA damage and, as a consequence, genome instability. The DNA damage response is a core signaling pathway in replicative stress. Our previous results on the proteome of HL-60 cells treated with ATRA suggest the involvement of major DNA repair regulators such as poly(ADP-ribose) polymerase 1 (PARP1) [11]. A recent study shows that mRNA translation and degradation play a critical role in response to genotoxic stress [32]. At the same time, changes related to endoplasmic reticulum stress could be associated with a putative regulatory mechanism involving components, which could be affected pharmacologically.

Transcriptomic analysis provides data on quantitative changes in regulatory molecules, e.g., TFs, which are components of signaling pathways regulating the balance between cell death and survival [33]. Such molecules have low abundance in cells, which complicates their analysis at the protein level [34].

The hierarchical modeling of the molecular response to the ATRA treatment results in the identification of particular regulatory molecules, thus significantly narrowing the search for potential new targets for the pharmacological action. In both ATRA-responsive cell lines, the transcriptional regulator Kaiso (ZBTB33) and the transcriptional repressor REST were determined as the TFs regulating DEGs. These TFs along with the corepressors SIN3A and RCOR1 are involved in the deacetylation and methylation of chromatin, thereby inhibiting the transcription of target genes.

The model schemes obtained for characterizing the process of the ATRA-induced differentiation of ATRA-responsive lines HL-60 and NB4 were based on transcriptomic and proteomic profiling data. For the HL-60 cell line, CDK6 has been found as a key molecule in the model scheme with down-regulated expression confirmed at both the proteomic and transcriptomic levels. CDK6 blocks myeloid differentiation by preventing *RUNX1* DNA binding and RUNX1-C/EBPα interaction [35]. In our previous study of the HL-60 nuclear proteome changes induced by the ATRA treatment, we found the TFs belonging to the SWI1/SNF1 and ARID families involved in the RUNX1-triggered pathway [36]. The pharmacological inhibition of CDK6 by palbociclib is one approach for the treatment of breast cancer [37]. Clinical studies are underway to investigate the efficacy of palbociclib in combination with dexamethasone, decitabine (an inhibitor of the DNA methyltransferase enzyme), or sorafenib (a multi-kinase inhibitor) in the treatment of AML [38]. The combination of therapy with the CDK6 inhibitor palbociclib and ATRA (tretinoin) may be an alternative approach for the treatment of AML.

For NB4 cells, TNF-alpha was identified in the current analysis as the key molecule at the transcriptome level. TNF-alpha is an inflammatory cytokine involved in every stage of leukemogenesis, from cellular transformation to proliferation, angiogenesis, and extramedullary infiltration [39]. Pharmacological antagonists of TNF-alpha are currently used for the treatment of autoimmune diseases [40]. It is possible that in combination with ATRA, they may be effective for the treatment of APL.

Changes in the content of proteins and transcripts registered in the experiment may be a consequence of earlier molecular events, not always associated with quantitative changes, for example, post-translational modifications. The importance of post-translational modifications for the antiproliferative and differentiating effect of antileukemic drugs has been shown by a number of studies. For example, HL-60 treated with HDAC inhibitors triggers the acetylation of proteins involved in cell cycle arrest and cytoskeleton remodeling [9]. In NB4 cells treated with ATRA combined with arsenic trioxide (ATO), a sustained demethylation of target genes (*RARβ* and *TGM2*) was found [41].

Based on results of transcriptomic and proteomic experiments, we have found pharmacologically relevant regulatory molecules, and the drug action on these targets will potentiate the antileukemic effect of ATRA

The scope of the current study was limited by omics experiments and bioinformatic predictions. To confirm the role of potential effectors in the response of leukemic cells to the ATRA treatment, biological validation (e.g., gene knockdown/overexpression experiments) needs to be conducted. The key molecules of modeling schemes CDK6 and TNF-alpha, as well as the most significantly altered DEGs and DEPs (e.g., BTG1, SPHK1, ANGPTL4), are thought to be the most promising targets for validation in vivo.

## 4. Materials and Methods

### 4.1. Cell Line Cultivation and the ATRA Treatment

HL-60 and K562 cell lines were provided by the cryobank “Collection of Vertebrate Cell Cultures” of the Institute of Cytology, Russian Academy of Sciences (St. Petersburg, Russia). NB4 cells were purchased from CLS Cell Lines Service GmbH (Eppelheim, Germany). The absence of mycoplasma was confirmed in the cell line passports and a DNA profile (STR) was provided. Cells not older than 10 passages were used in the experiments and were regularly checked for the absence of mycoplasma.

Before omics experiments, we performed an MTT assay. The cytotoxicity of ATRA added in a dosage of 0–100 μM against all three cell lines was studied at 72 h after the ATRA exposure. A dose of a substance that causes a 10–30 percent decrease in cell viability is generally considered subtoxic [42,43]. In our experiment, the dosage of 10 μM resulted in a 20% decrease in cell viability. The further omics experiments were conducted under the 10 μM ATRA treatment.

The cells of HL-60, NB4, and K562 lines were cultivated in RPMI-1640 growth medium supplemented with 10% FBS (*v*/*v*), 100 U/mL of penicillin, 100 U/mL of streptomycin, and 2 mM L-glutamine (all Gibco™, Paisley, UK) in a CO_2_ incubator under standard conditions (37 °C, 5% CO_2_, 80% humidity). ATRA (Sigma Aldrich, St. Louis, MO, USA) was added to reach a final concentration of 10 μM to all cell cultures.

In our previous studies, we applied different time point schedules to the time-course proteomic (i.e., 0, 3, 24, 48, and 96 h, and 0, 3, 6, 9, 12, and 72 h) and transcriptomic (0, 3, 24, and 96 h) [11,36] investigation of leukemic cells under the ATRA treatment. In the current study, we used unified schedules for both transcriptomic and proteomic analysis. These time schemes combined early time points 3 h and 12 h that allowed the first molecular events of the cell response to the ATRA treatment to be elucidated. We also included advanced time points, i.e., 24 h and 72 h, when granulocyte-specific surface markers CD11b and CD38 could be detected by cytofluorometry, as was shown previously [26].

The differentiation was performed in two biological replicates for both proteomic and transcriptomic experiments. For the proteome and transcriptome profiling of HL-60 and NB4 cell lines, the cells were harvested at 0 h, 3 h, 12 h, 24 h, and 72 h after the ATRA addition. To analyze the proteome and transcriptome of K562 cells, the cells were harvested at 0 h, 24 h, and 72 h after the ATRA addition.

### 4.2. Measurement of the CD Marker Profile Using Flow Cytometry

Cell lines HL60, NB4, and K562 were seeded in 2 mL in six-well plates at a concentration of 0.5 × 106 cells/mL in RPMI-1640 medium with 10% FBS, GlutaMAXTM, and 1% penicillin/streptomycin. ATRA was then added to each well to reach the final concentration of 10 μM. Untreated cells were used as a control (C). After 72 h of exposure to ATRA, cells were separated from the supernatant by centrifugation at 300× *g* for 5 min. Cells were washed twice with phosphate-buffered saline (PBS) (pH 7.4) and fixed in 4% formaldehyde in PBS for 1 h. The cells were then washed twice in PBS, and nonspecific binding was blocked for 20 min by incubation in a 1% solution of bovine serum albumin (BSA) in PBS. FITC- and APC-conjugated antibodies to CD11b and CD38, respectively, were diluted according to the manufacturer’s instructions in 1% bovine serum albumin in PBS (pH 7.4) and 100 μL aliquots were used to stain one sample (1 million cells). Unstained cells were examined as an isotype control (IC). Applying flow cytometry, granulocytic differentiation was assessed by the CD11b and CD38 expression levels using the ZE5 Cell Analyzer (BioRad, Hercules, CA, USA). Analysis of the results and visualization were carried out using the Floreada.io software (https://floreada.io/analysis, accessed on 20 November 2023).

### 4.3. Transcriptomic Analysis

Total RNA was isolated using an RNeasy Mini Kit (QIAGEN, Hilden, Germany) according to the manufacturer’s recommendations. The quality of the resulting RNA was determined on an Agilent 2100 Bioanalyser instrument using RNA 6000 Nano LabChip chips (all Agilent Technologies, Santa Clara, CA, USA). For further analysis, only samples with an RIN (RNA Integrity Number) of at least 8 were used. The RNA concentration and efficiency of fluorescent label incorporation were determined spectrophotometrically on a NanoDrop ND-1000 instrument (Thermo Fisher Scientific, Waltham, MA, USA). RNA preparation for hybridization was performed using a LowInput QuickAmp Labeling Kit (Agilent Technologies, Santa Clara, CA, USA) according to the One-Color Microarray-Based Gene Expression Analysis protocol (version 6.9.1). Since the analysis was carried out in a single-color hybridization format, fluorescently labeled 3-CTP nucleotides (PerkinElmer, Waltham, MA, USA) were used as a label. Also, according to the standard protocol, appropriate amounts of RNA from the Agilent RNA Spike-In Kit (Agilent Technologies, Santa Clara, CA, USA) were added to the samples. Fluorescently labeled cRNA was fragmented, hybridized with 4x44K G4112F Agilent whole-genome expression chips, and scanned on an Agilent G2505B confocal laser scanner (all Agilent Technologies, Santa Clara, CA, USA). Data extraction and primary statistical processing were performed using Feature Extraction 10.10.1.1 software (Agilent Technologies, Santa Clara, CA, USA).

All processing steps were executed with the R-package “limma”. Background correction on the fluorescent intensities was performed using the” backgroundCorrect” function with “normexp” as the method followed by normalization by the “normalizedBetweenArray” function using the “quantile” method. The intensity values were log2-transformed. Control probes and probes with intensities below background noise were filtered out. Values for within-array duplicate probes are replaced with their average using the “avereps” function by “Probe name” and “GeneName” columns. Data were normalized separately for HL-60, NB4, and K562 cell lines. The *p*-value was adjusted with a Benjamini–Hochberg (BH) multiple testing correction. The transcriptomic data were deposited into the ArrayExpress repository through the Annotare submission system (v. 2.0, EMBL-EBI, Hinxton Cambridge, UK). The data were uploaded to the BioStudies repository and available with accession number E-MTAB-13723.

### 4.4. Sample Preparation Prior Mass-Spectrometry

To prepare samples for mass spectrometric analysis, 200 μL of lysis buffer containing 1% SDS and a cocktail of protease inhibitors cOmplete™ and Mini Protease Inhibitor Cocktail (Sigma-Aldrich, St. Louis, MO, USA) in 100 mM Tris HCl (pH 8.5) were added to each sample. The samples were sonicated using an ultrasonic disintegrator with a Bandelin Sonopuls (Bandelin Electronic, Berlin, Germany) probe at 50% power for 1 min in ice. After this, the samples were centrifuged for 15 min at a speed of 14,000× *g* at a temperature of 10 °C. The supernatants were used for subsequent trypsinolysis. The concentration of total protein in the resulting samples was determined by a colorimetric method using a commercial Pierce TM BCA Protein Assay Kit (Thermo Fisher Scientific, Waltham, MA, USA) in accordance with the manufacturer’s recommendations.

The hydrolysis of proteins was carried out according to the FASP Protocol (Filter-Aided Sample Preparation) with slight modification. Briefly, each sample in an amount of 100 µg was transferred to concentration filters with a cut-off of 30 kDa (Merck Millipore Limited, Tullagree, Ireland) by centrifugation at 11,000× *g* for 15 min at 20 °C. To break the disulfide bonds, each sample was incubated with 30 mM tris(2-carboxyethyl)phosphine (TCEP, Thermo Fisher Scientific, Waltham, MA, USA) and 50 mM 2-chloroacetamide (CAA, Sigma-Aldrich, St. Louis, MO, USA) at 80 °C for 1 h. Then, the samples were washed 3 times with a buffer containing 8 M urea (Sigma-Aldrich, St. Louis, MO, USA) in 100 mM Tris HCl, pH 8.5, and washed twice with 50 mM triethylammonium bicarbonate buffer (TEAB, Sigma-Aldrich, St. Louis, MO, USA), pH 8.5, by centrifugation at 11,000× *g* for 15 min at 20 °C. Then, 50 µL of 50 mM TEAB (Sigma-Aldrich, St. Louis, MO, USA), pH 8.5, and trypsin (Promega, Fitchburg, WI, USA) at a “trypsin to total protein” ratio of 1:70 was added. The samples were incubated overnight at 37 °C. After incubation, the peptides were eluted by centrifugation at 11,000× *g* for 15 min at 20 °C, and the filter was washed twice with 50 µL of 5% formic acid (Sigma-Aldrich, St. Louis, MO, USA). The peptide concentration was determined by the colorimetric method using a Pierce Quantitative Colorimetric Peptide Assay kit (Pierce, Rockford, IL, USA) in accordance with the manufacturer’s recommendations. The peptides were dried and dissolved in 0.1% formic acid (Sigma-Aldrich, St. Louis, MO, USA) to a final concentration of 3 µg/µL. To check sample loading prior to TMT-labeling, the resultant samples were analyzed by high-resolution mass spectrometry.

### 4.5. Samples Isobaric Mass Tag Labeling

For TMT-labeling, 5 μg of peptides of each sample (pre-dried in a vacuum concentrator) was reconstituted in 100 mM TEAB (Sigma-Aldrich, St. Louis, MO, USA), pH 8.5, to a concentration of 0.2 μg/μL. TMT-10 reagents (Thermo Fisher Scientific, Waltham, MA, USA) were resuspended in anhydrous ACN to a concentration of 19.5 μg/μL. The appropriate TMT reagent was added to each sample at 1:35 reagent/peptide (*wt*/*wt*) and incubated for 1 h at room temperature. To quench the reaction, 5% hydroxylamine (Sigma-Aldrich, St. Louis, MO, USA) was added and samples were incubated for 15 min at room temperature. Labeled samples that corresponded to the time points 0 h, 12 h, 24 h, and 72 h were combined within a biological repeat for each cell line, resulting in 6 samples for MS/MS analysis. Six pooled samples were dried via vacuum centrifugation and desalted using a C18 Stage-Tip (Thermo Fisher Scientific, Waltham, MA, USA) method. The peptides were dissolved in 0.1% formic acid (Sigma-Aldrich, St. Louis, MO, USA) to the final concentration of 3 µg/µL.

### 4.6. Shotgun Mass-Spectrometry

The peptide samples obtained were analyzed using the Ultimate (Thermo Scientific, Waltham, MA, USA) connected to a Q Exactive HF-X Quadrupole-Orbitrap, equipped with a nanoelectrospray ion source (Thermo Scientific, Waltham, MA, USA). Peptide separations were carried out on an RP-HPLC Zorbax 300SBC18 column (C18 3.5 µm, 75 µm inner diameter and 150 mm length, Agilent Technologies, Santa Clara, CA, USA) using a linear gradient from 98% solvent A (water, 0.1% formic acid) and 2% solvent B (water, 0.1% formic acid, and 80% acetonitrile) to 30% solvent B over 90 min at a flow rate of 0.3 µL/min. Mass spectra were acquired in the positive ion mode using an Orbitrap analyzer with a resolution of 120,000 (*m*/*z* = 400) for MS and 30,000 (*m*/*z* = 400) for MS/MS scans. The AGC target was set at 1 × 10^6^ and 2 × 10^5^ with a maximum ion injection time of 50 ms and 100 ms for MS and MS/MS, respectively. The survey MS scan was followed by MS/MS spectra for 40 of the most abundant precursors. The higher-energy collisional dissociation (HCD) was used, and the normalized collision energy was set to 29. The signal threshold was set to 50,000 for an isolation window of 1 *m*/*z*. The fragmented precursors were dynamically excluded from targeting with a repeat count of 1, repeat duration of 10 s, and exclusion duration of 60 s. Singly charged ions and those without a defined charge state were excluded from triggering the MS/MS scans. Dionex™ Cytochrome C Digest (Thermo Scientific, Waltham, MA, USA) was used for quality control LC-MS/MS runs.

For identification and TMT-based quantification, mass spectrometry data were loaded into the MaxQuant software (version 2.0.3.0). Proteins were identified using the built-in Andromeda algorithm. Identification was carried out using the FASTA file (UP000005640, UniProt Release 2022_02, 20,598 proteins, EMBL-EBI, Hinxton Cambridge, UK) and its inverted counterpart to calculate the frequency of false positive identifications (FDR), alongside a built-in database of potential contaminants. The carbamidomethylation of cysteine was used as a fixed modification, and methionine oxidation and N-terminal acetylation were used for variable modification. Trypsin was selected as the protease, and two missed cleavages were allowed. The tolerance for the precursor and fragment ions was 20 ppm. For proteins and peptides, the FDR threshold value was 0.01. Quantitative analysis was carried out on the basis of the reporter ion (MS2) intensity value using the algorithm built into MaxQuant (version 2.0.3.0). All working channels were assigned as a reference and used for weighted median normalization. Matching between-run (MBR) functions were applied.

The summed signal for all proteins in each TMT channel was calculated, and the TMT channel with the highest summed signal was determined. Normalization factors that represent the ratio of the highest summed signal to the summed signal of each TMT channel were calculated. Sample loading was corrected through multiplication of the reporter ion intensity for each protein by the TMT-channel-specific normalization factor.

### 4.7. Upstream Regulator Search in GeneXplain Platform

GeneXplain platform 2023.1 software packages (http://platform.genexplain.com, accessed on 10 December 2023) and the TRANSFAC^®^ database were used to search for over-represented transcription factor binding sites (TFBS) [44]. Lists of DEGs at the 3 h, 12 h, 24 h, and 72 h time points of the ATRA treatment (Genes: Gene Symbol as an identifier) obtained for HL-60 and NB4, and lists of DEGs at the 24 h and 72 h time points of the ATRA treatment obtained for K562 cells, were used for analysis. For the mapping to model patterns, we used the lists of DEPs and MS-identified proteins for each cell line, which were previously converted from “Genes: Gene Symbol identifiers” into “Proteins identifiers: Protein Transpath”.

The search for the over-represented TF binding sites in the promoter regions of genes corresponding to the transcripts of the test set (DEGs at 3 h, 12 h, 24 h, and 72 h time points of the ATRA treatment of HL-60 and NB4 cells; DEGs at 24 h and 72 h time points of the ATRA treatment of K562 cells) was conducted using the “Site search on gene”, “module set”, and the TRANSFAC^®^ database. The search region in the promoter region was in the range from −1000 to +100 bp from the transcription start site, and only the most confirmed promoters were used for analysis. During the analysis, the frequencies of occurrence of TRANSFAC^®^ database matrices corresponding to TF binding sites in the promoters of genes encoding proteins of the test set were compared with those of genes encoding proteins of control samples (background) at each time point. For the ratio of the frequency of occurrence of TF binding sites in the test and control samples, the threshold was >1.4 with a *p*-value < 0.005. The matrices were converted into a set of TFs (using the identifier “Protein Tranpath peptides”), which were in turn annotated using the identifier “Gene Symbol”. The matrices of TFs obtained for DEGs at 3, 12, 24, and 72 h after the ATRA treatment were combined into one list within each cell line studied. For the set of TFs obtained in the previous stage, a search for a common regulator was performed using the “Regulator search” module of the geneXplain platform (http://platform.genexplain.com, accessed on 10 December 2023) with the following settings: database used, TRANSPATH; path length, R = 10; and cut-off of results by FDR, 0.01. For each potential key regulator, in addition to FDR, Score, Z-score, and Ranks sum were calculated.

### 4.8. Targeted Mass-Spectrometry Analysis

The content of proteotypic peptides VELAPLPSWQPVGK and ASVSVTAEDEGTQR mapped to the ICAM1 protein, as well as the content of proteotypic peptide VLELTAENER mapped to the CEBPB protein, was measured. The experimental samples of all cell lines studied that were obtained at 0, 24, and 72 h in replicates were subjected to SRM/SIS analysis. The same samples were used for the TMT-based shotgun mass spectrometric analysis.

Solid-phase synthesis of the SIS peptides was carried out in-house with an Overture automated peptide synthesizer (Protein Technologies, Manchester, UK), according to the published method [45]. Isotopically labeled amino acids Fmoc-Lys-OH-(13C6, 15N2) and Fmoc-Arg-OH (13C6, 15N2) (Cambridge Isotope Laboratories, Tewksbury, MA, USA) were used for the synthesis of isotopically labeled peptides instead of conventional lysine and arginine. SIS peptides were spiked into experimental samples to a final quantity of 50 fmol/μg of total protein.

All experimental samples were analyzed in three technical repeats. Chromatographic separation was performed in a micro-flow mode with an Agilent 1200 series system (Agilent Technologies, Santa Clara, CA, USA) connected to a TSQ Quantiva triple quadrupole mass analyzer (Thermo Scientific, Waltham, MA, USA). A 3 μL sample containing 15 μg of native peptides and SIS peptides was separated on a ZORBAX SB-C18 analytical column (150 × 0.5 mm; particle size, 5 μm; Agilent Technologies, Santa Clara, CA, USA) in a linear gradient of acetonitrile concentration at a flow rate of 20 µL/min. The LC-SRM analysis was performed as described previously [36]. All transitions with recorded signals were used for quantification with the Skyline software (version 4.1.0, University of Washington, Seattle, WA, USA). The transitions and normalized collision energy values (V) are listed in Table S3.

## 5. Conclusions

Proteomic and transcriptomic methods provide a large amount of data on differentially expressed proteins and RNAs in leukemia cells exposed to the influence of differentiating and antitumor agents. In the current study, the ATRA-responsive cell lines HL-60 and NB4 and the ATRA-non-responsive cell line K562 were subjected to transcriptome and proteome profiling in a time-course manner. The approach we have described in this study, combining transcriptomic and proteomic profiling with the upstream regulator search and hierarchical modeling, can be applied to the study of the molecular response to substances, including ATRA, both in stable cell lines and in primary cell cultures. Taking into consideration that high genetic heterogeneity is a major challenge in AML treatment, this approach could be used as a tool for additional drug target search and, as a consequence, for the development of customer-tailored treatment. Notably, our results indicate that potential regulatory molecules may be targets of already-approved anticancer drugs used to treat other types of cancer (e.g., Palbociclib). This is consistent with the recently popular concept of drug repositioning.

## Data Availability

The mass spectrometry proteomics data have been deposited in the ProteomeXchange Consortium via the PRIDE partner repository with the dataset identifier PXD050317. The transcriptomic data were uploaded to the BioStudies repository and are available with accession number E-MTAB-13723.

## Supplementary Materials

The following sporting information can be downloaded at https://www.mdpi.com/article/10.3390/ijms25094618/s1.

## References

[1] Brunning R.D. (2003). Classification of Acute Leukemias. Semin. Diagn. Pathol..

[2] Kantarjian H., Kadia T., DiNardo C., Daver N., Borthakur G., Jabbour E., Garcia-Manero G., Konopleva M., Ravandi F. (2021). Acute Myeloid Leukemia: Current Progress and Future Directions. Blood Cancer J..

[3] Segalla S., Rinaldi L., Kilstrup-Nielsen C., Badaracco G., Minucci S., Pelicci P.G., Landsberger N. (2003). Retinoic Acid Receptor Alpha Fusion to PML Affects Its Transcriptional and Chromatin-Remodeling Properties. Mol. Cell. Biol..

[4] Lonetti A., Iacobucci I., Masetti R. (2019). Successes and Challenges for Diagnosis and Therapy of Acute Leukemia. J. Oncol..

[5] Yeoh Z.H., Bajel A., Wei A.H. (2021). New Drugs Bringing New Challenges to AML: A Brief Review. J. Pers. Med..

[6] Sallman D.A., DeZern A.E., Garcia-Manero G., Steensma D.P., Roboz G.J., Sekeres M.A., Cluzeau T., Sweet K.L., McLemore A., McGraw K.L. (2021). Eprenetapopt (APR-246) and Azacitidine in TP53-Mutant Myelodysplastic Syndromes. J. Clin. Oncol. Off. J. Am. Soc. Clin. Oncol..

[7] Tasseff R., Jensen H.A., Congleton J., Dai D., Rogers K.V., Sagar A., Bunaciu R.P., Yen A., Varner J.D. (2017). An Effective Model of the Retinoic Acid Induced HL-60 Differentiation Program. Sci. Rep..

[8] Ueda M., Stefan T., Stetson L., Ignatz-Hoover J.J., Tomlinson B., Creger R.J., Cooper B., Lazarus H.M., de Lima M., Wald D.N. (2020). Phase I Trial of Lithium and Tretinoin for Treatment of Relapsed and Refractory Non-Promyelocytic Acute Myeloid Leukemia. Front. Oncol..

[9] Zhu X., Liu X., Cheng Z., Zhu J., Xu L., Wang F., Qi W., Yan J., Liu N., Sun Z. (2016). Quantitative Analysis of Global Proteome and Lysine Acetylome Reveal the Differential Impacts of VPA and SAHA on HL60 Cells. Sci. Rep..

[10] Albanesi J., Noguera N.I., Banella C., Colangelo T., De Marinis E., Leone S., Palumbo O., Voso M.T., Ascenzi P., Nervi C. (2020). Transcriptional and Metabolic Dissection of ATRA-Induced Granulocytic Differentiation in NB4 Acute Promyelocytic Leukemia Cells. Cells.

[11] Novikova S., Tikhonova O., Kurbatov L., Farafonova T., Vakhrushev I., Lupatov A., Yarygin K., Zgoda V. (2021). Omics Technologies to Decipher Regulatory Networks in Granulocytic Cell Differentiation. Biomolecules.

[12] Thatcher J.E., Isoherranen N. (2009). The Role of CYP26 Enzymes in Retinoic Acid Clearance. Expert Opin. Drug Metab. Toxicol..

[13] Stevison F., Hogarth C., Tripathy S., Kent T., Isoherranen N. (2017). Inhibition of the All-Trans Retinoic Acid (at RA) Hydroxylases CYP26A1 and CYP26B1 Results in Dynamic, Tissue-Specific Changes in Endogenous at RA Signaling. Drug Metab. Dispos..

[14] Russo D., Regazzi M., Sacchi S., Visani G., Lazzarino M., Avvisati G., Pelicci P.G., Dastoli G., Grandi C., Iacona I. (1998). All-Trans Retinoic Acid (ATRA) in Patients with Chronic Myeloid Leukemia in the Chronic Phase. Leukemia.

[15] SUN D., WANG S. (2021). Sphingosine Kinases Are Involved in the Regulation of All-Trans Retinoic Acid Sensitivity of K562 Chronic Myeloid Leukemia Cells. Oncol. Lett..

[16] Yuniati L., Scheijen B., van der Meer L.T., van Leeuwen F.N. (2019). Tumor Suppressors BTG1 and BTG2: Beyond Growth Control. J. Cell. Physiol..

[17] Xue K., Wu J.C., Li X.Y., Li R., Zhang Q.L., Chang J.J., Liu Y.Z., Xu C.H., Zhang J.Y., Sun X.J. (2021). Chidamide Triggers BTG1-Mediated Autophagy and Reverses the Chemotherapy Resistance in the Relapsed/Refractory B-Cell Lymphoma. Cell Death Dis..

[18] Gupta P., Mohammad T., Dahiya R., Roy S., Noman O.M.A., Alajmi M.F., Hussain A., Hassan M.I. (2019). Evaluation of Binding and Inhibition Mechanism of Dietary Phytochemicals with Sphingosine Kinase 1: Towards Targeted Anticancer Therapy. Sci. Rep..

[19] Gupta P., Taiyab A., Hussain A., Alajmi M.F., Islam A., Hassan M.I. (2021). Targeting the Sphingosine Kinase/Sphingosine-1-Phosphate Signaling Axis in Drug Discovery for Cancer Therapy. Cancers.

[20] Yoo H.S., Cockrum M.A., Napoli J.L. (2023). Cyp26a1 Supports Postnatal Retinoic Acid Homeostasis and Glucoregulatory Control. J. Biol. Chem..

[21] Brown G. (2023). Targeting the Retinoic Acid Pathway to Eradicate Cancer Stem Cells. Int. J. Mol. Sci..

[22] Su M., Alonso S., Jones J.W., Yu J., Kane M.A., Jones R.J., Ghiaur G. (2015). All-Trans Retinoic Acid Activity in Acute Myeloid Leukemia: Role of Cytochrome P450 Enzyme Expression by the Microenvironment. PLoS ONE.

[23] Li X., Chen T., Shi Q., Li J., Cai S., Zhou P., Zhong Y., Yao L. (2015). Angiopoietin-like 4 Enhances Metastasis and Inhibits Apoptosis via Inducing Bone Morphogenetic Protein 7 in Colorectal Cancer Cells. Biochem. Biophys. Res. Commun..

[24] Fang Y., Li X., Cheng H., Zhang L., Hao J. (2022). ANGPTL4 Regulates Lung Adenocarcinoma Pyroptosis and Apoptosis via NLRP3\ASC\Caspase 8 Signaling Pathway to Promote Resistance to Gefitinib. J. Oncol..

[25] Zheng J., Umikawa M., Cui C., Li J., Chen X., Zhang C., Hyunh H., Kang X., Silvany R., Wan X. (2012). Inhibitory Receptors Bind ANGPTLs and Support Blood Stem Cells and Leukaemia Development. Nature.

[26] Novikova S.E., Tolstova T.V., Soloveva N.A., Farafonova T.E., Tikhonova O.V., Kurbatov L.K., Rusanov A.L., Zgoda V.G. (2023). System Analysis of Surface CD Markers during the Process of Granulocytic Differentiation. Biomed. Khim..

[27] Liu Y., Zhang J., Du Z., Huang J., Cheng Y., Yi W., Li T., Yang J., Chen C. (2023). Comprehensive Analysis of PTPN Family Expression and Prognosis in Acute Myeloid Leukemia. Front. Genet..

[28] Schneider C., Oellerich T., Baldauf H.-M., Schwarz S.-M., Thomas D., Flick R., Bohnenberger H., Kaderali L., Stegmann L., Cremer A. (2017). SAMHD1 Is a Biomarker for Cytarabine Response and a Therapeutic Target in Acute Myeloid Leukemia. Nat. Med..

[29] Lin Y.-T., Chao C.C.K. (2015). Identification of the SS-Catenin/JNK/Prothymosin-Alpha Axis as a Novel Target of Sorafenib in Hepatocellular Carcinoma Cells. Oncotarget.

[30] Lv C., Huang Y., Wang Q., Wang C., Hu H., Zhang H., Lu D., Jiang H., Shen R., Zhang W. (2023). Ainsliadimer A Induces ROS-Mediated Apoptosis in Colorectal Cancer Cells via Directly Targeting Peroxiredoxin 1 and 2. Cell Chem. Biol..

[31] Kaida A., Iwakuma T. (2021). Regulation of P53 and Cancer Signaling by Heat Shock Protein 40/J-Domain Protein Family Members. Int. J. Mol. Sci..

[32] Mohanan G., Das A., Rajyaguru P.I. (2021). Genotoxic Stress Response: What Is the Role of Cytoplasmic MRNA Fate?. Bioessays.

[33] Horta M.A.C., Pimenta R.J.G., Almeida D.A., Rosolen R.R., Aono A.H., Filho J.F., de Oliveira F.A., Niederauer G.F., Ferreira R.C.U., Bajay S.K. (2023). Transcriptomic Analysis of Genes: Expression and Regulation. Transcriptome Profiling Progress and Prospects.

[34] Jiang D., Jarrett H.W., Haskins W.E. (2009). Methods for Proteomic Analysis of Transcription Factors. J. Chromatogr. A.

[35] Fujimoto T., Anderson K., Jacobsen S.E.W., Nishikawa S.I., Nerlov C. (2007). Cdk6 Blocks Myeloid Differentiation by Interfering with Runx1 DNA Binding and Runx1-C/EBPalpha Interaction. EMBO J..

[36] Novikova S., Tolstova T., Kurbatov L., Farafonova T., Tikhonova O., Soloveva N., Rusanov A., Archakov A., Zgoda V. (2022). Nuclear Proteomics of Induced Leukemia Cell Differentiation. Cells.

[37] Turner N.C., Ro J., André F., Loi S., Verma S., Iwata H., Harbeck N., Loibl S., Huang Bartlett C., Zhang K. (2015). Palbociclib in Hormone-Receptor-Positive Advanced Breast Cancer. N. Engl. J. Med..

[38] Uras I.Z., Sexl V., Kollmann K. (2020). CDK6 Inhibition: A Novel Approach in AML Management. Int. J. Mol. Sci..

[39] Zhou X., Li Z., Zhou J. (2017). Tumor Necrosis Factor α in the Onset and Progression of Leukemia. Exp. Hematol..

[40] Koutnik-Fotopoulos E. (2012). TNF-α Antagonist Use in Autoimmune Diseases. First Rep. Manag. Care.

[41] Huynh T.T., Sultan M., Vidovic D., Dean C.A., Cruickshank B.M., Lee K., Loung C.Y., Holloway R.W., Hoskin D.W., Waisman D.M. (2019). Retinoic Acid and Arsenic Trioxide Induce Lasting Differentiation and Demethylation of Target Genes in APL Cells. Sci. Rep..

[42] Romashin D., Arzumanian V., Poverennaya E., Varshaver A., Luzgina N., Rusanov A. (2024). Evaluation of Cd-Induced Cytotoxicity in Primary Human Keratinocytes. Hum. Exp. Toxicol..

[43] Paesano L., Perotti A., Buschini A., Carubbi C., Marmiroli M., Maestri E., Iannotta S., Marmiroli N. (2017). Data on HepG2 Cells Changes Following Exposure to Cadmium Sulphide Quantum Dots (CdS QDs). Data Br..

[44] Matys V., Kel-Margoulis O.V., Fricke E., Liebich I., Land S., Barre-Dirrie A., Reuter I., Chekmenev D., Krull M., Hornischer K. (2006). TRANSFAC and Its Module TRANSCompel: Transcriptional Gene Regulation in Eukaryotes. Nucleic Acids Res..

[45] Hood C.A., Fuentes G., Patel H., Page K., Menakuru M., Park J.A.E.H. (2008). Fast Conventional Fmoc Solid-Phase Peptide Synthesis with HCTU. J. Pept. Sci. Off. Publ. Eur. Pept. Soc..

