# Supplementary material for: Systems Biology for Drug Target Discovery in Acute Myeloid Leukemia
Source: Int J Mol Sci. 2024 Apr 23;25(9):4618. doi: 10.3390/ijms25094618 (PMC11083274; doi:10.3390/ijms25094618)
Supplement: Supplementary file 1 [file ijms-25-04618-s001.zip › Supplementary materials.pdf]

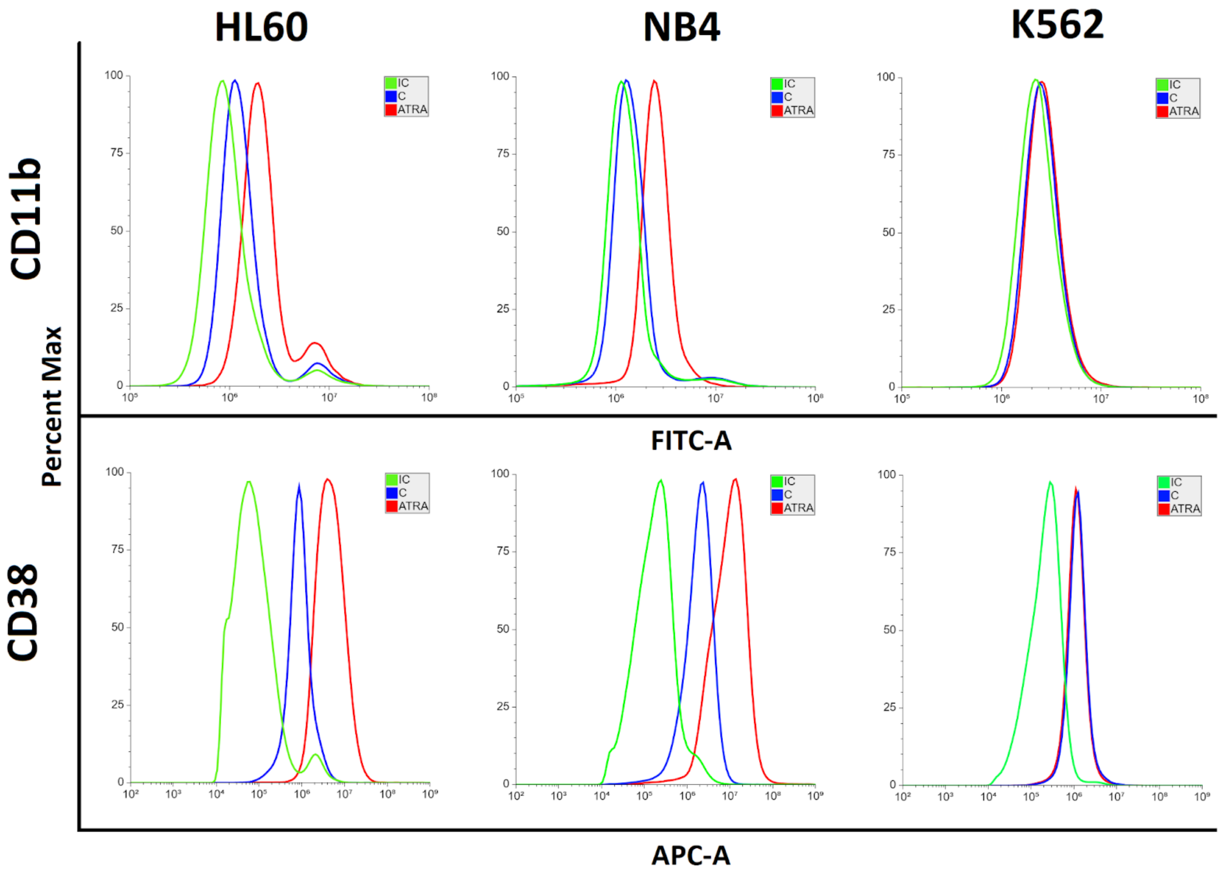

Figure S1. Flow cytometric assessment of the granulocytic markers CD11b and CD38 surface expression in HL-60, NB4, and K562 cells at 72 h after the all-*trans*-retinoid acid (ATRA) treatment (red), compared to non-treated cells (blue).

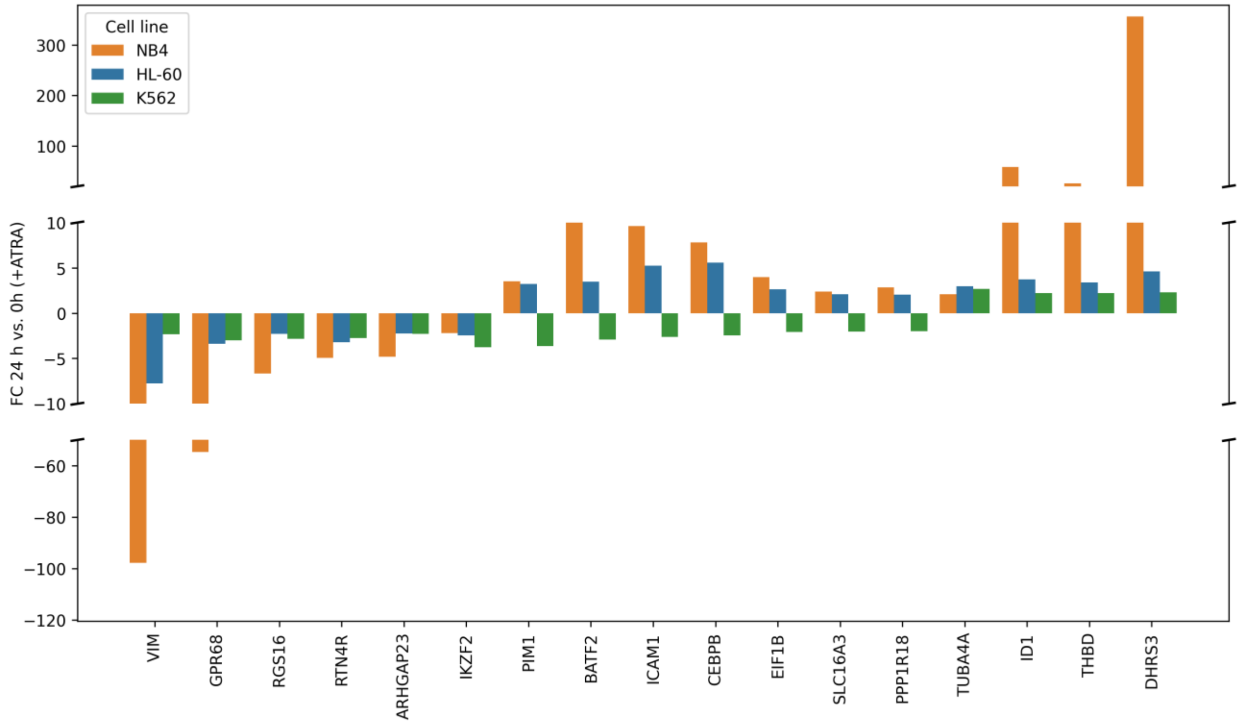

Figure S2. The transcriptomic profile of 17 differentially expressed genes (DEGs) that are common for ATRA-non-responsive cell line K562 and ATRA-responsive cell lines NB4 and HL-60 at the 24 h after the ATRA treatment compared to control (0 h). FC - fold change (adjusted p-value <0.05).

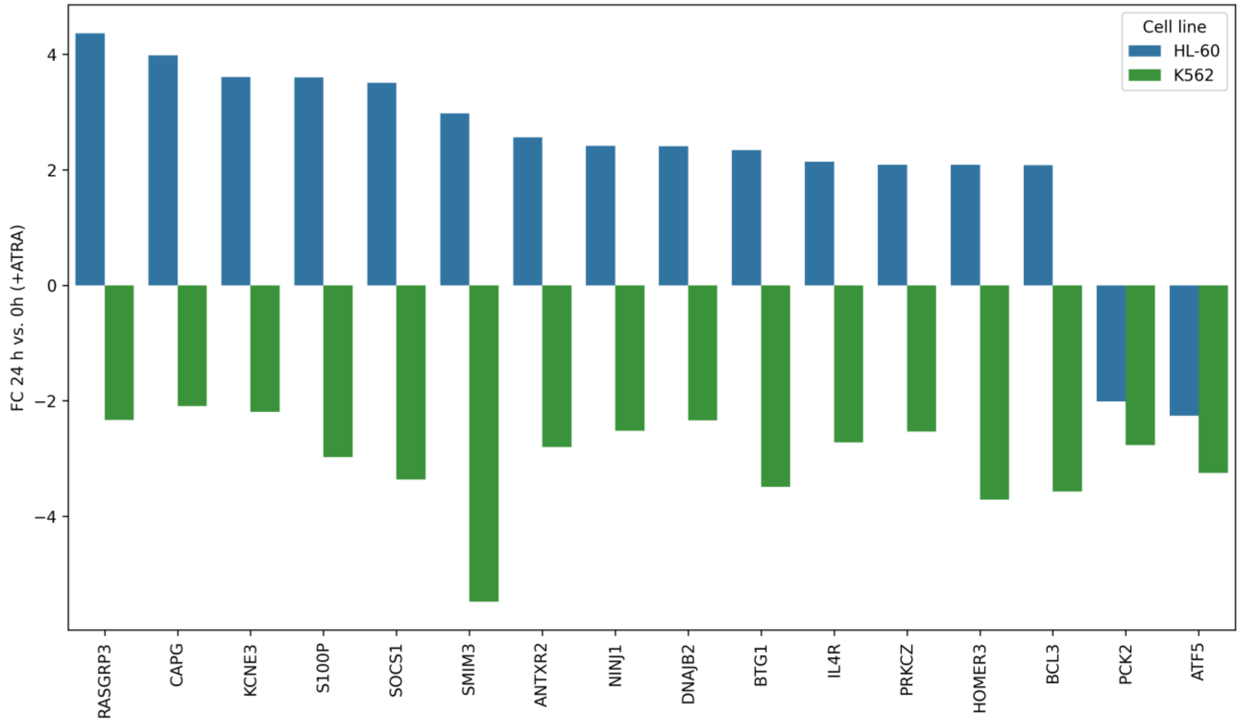

Figure S3. The transcriptomic profile of 16 differentially expressed genes (DEGs) that are common for ATRA-non-responsive cell line K562 and ATRA-responsive HL-60 cell line at the 24 h after the ATRA treatment compared to control (0 h). FC - fold change (adjusted p-value <0.05).

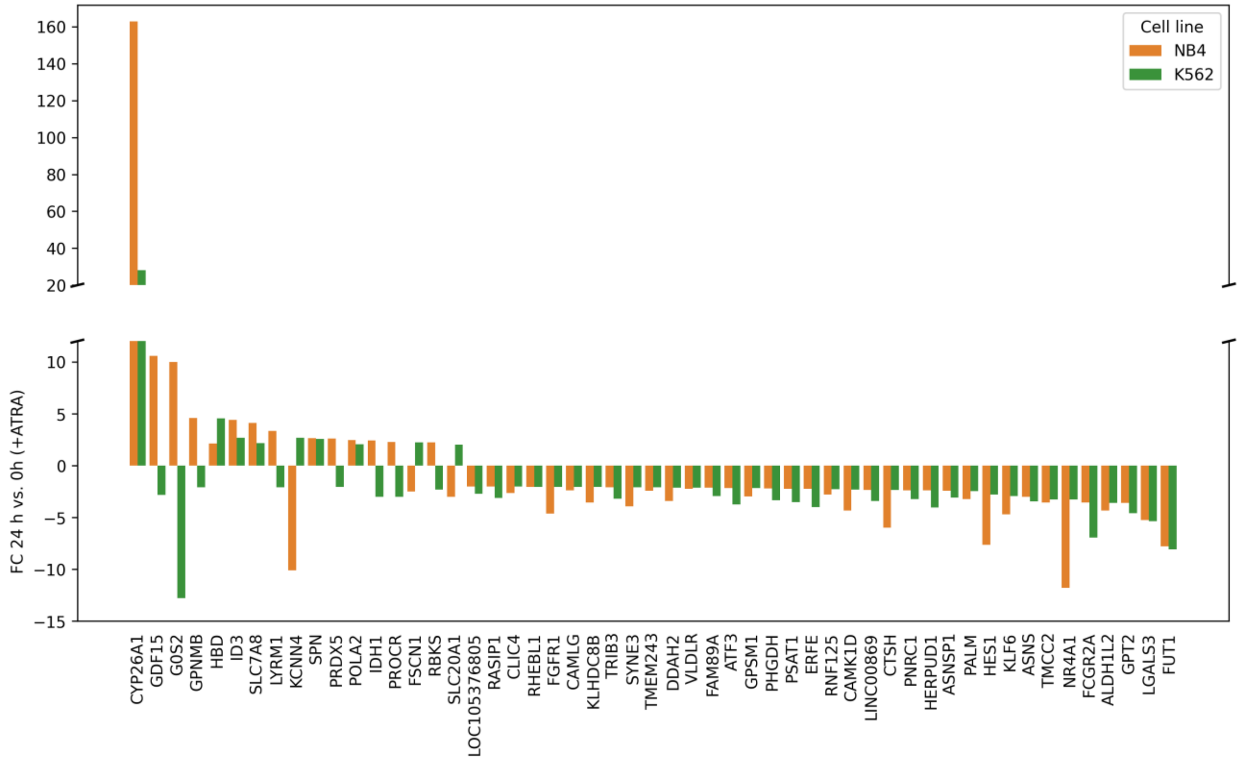

Figure S4. The transcriptomic profile of 52 differentially expressed genes (DEGs) that are common for ATRA-non-responsive cell line K562 and ATRA-responsive NB4 cell line at the 24 h after the ATRA treatment compared to control (0 h). FC - fold change (adjusted p-value <0.05).

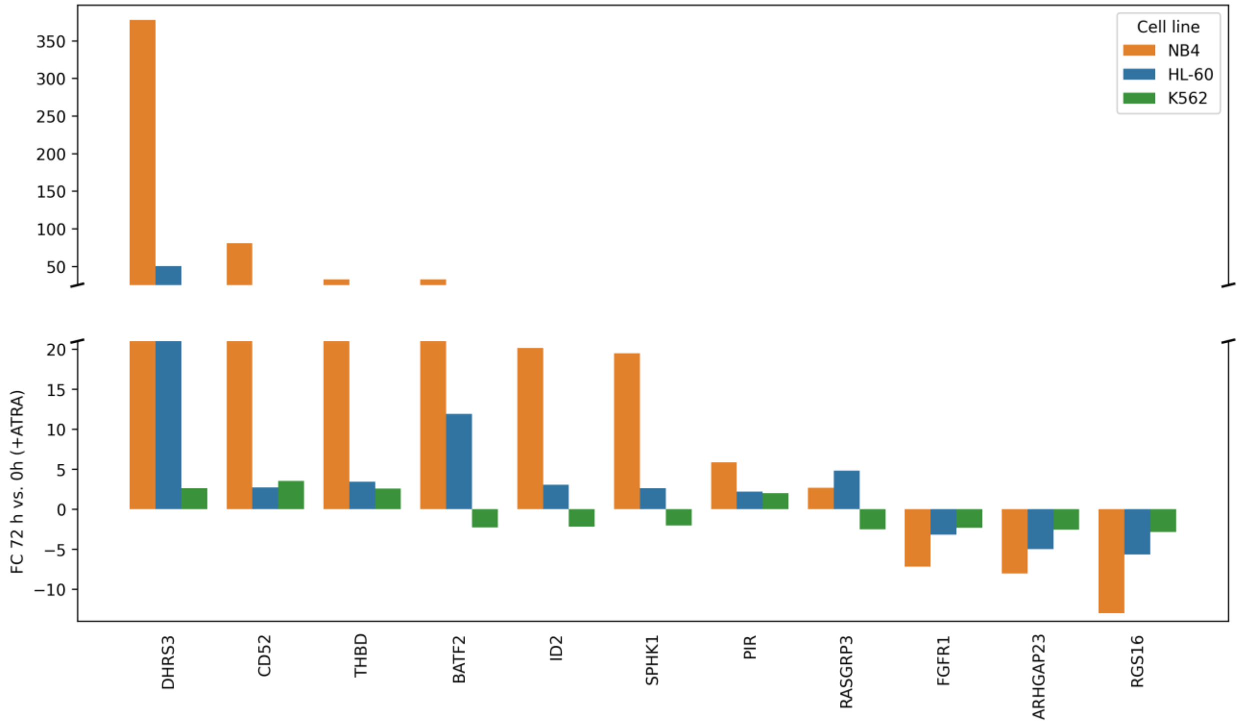

Figure S5. The transcriptomic profile of 11 differentially expressed genes (DEGs) that are common for ATRA-non-responsive cell line K562 and ATRA-responsive cell lines NB4 and HL-60 at the 72 h after the ATRA treatment compared to control (0 h). FC - fold change (adjusted p-value <0.05).

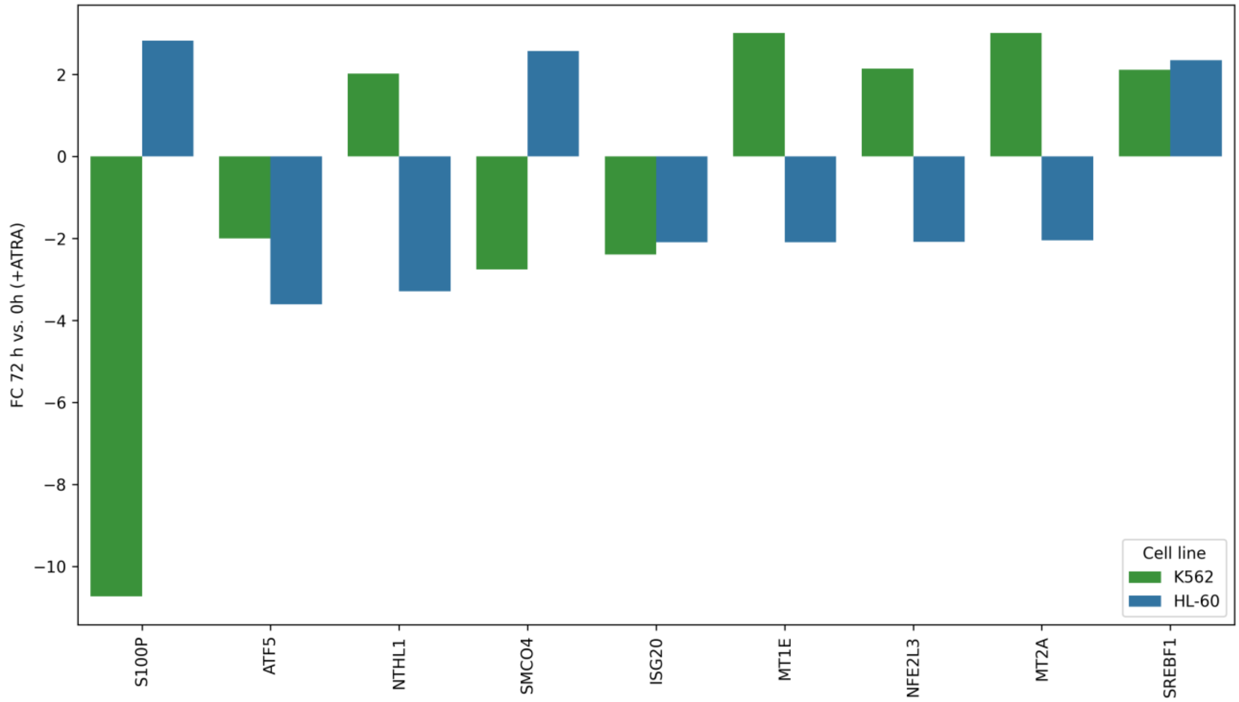

Figure S6. The transcriptomic profile of 9 differentially expressed genes (DEGs) that are common for ATRA-non-responsive cell line K562 and ATRA-responsive HL-60 cell line at the 72 h after the ATRA treatment compared to control (0 h). FC - fold change (adjusted p-value <0.05).

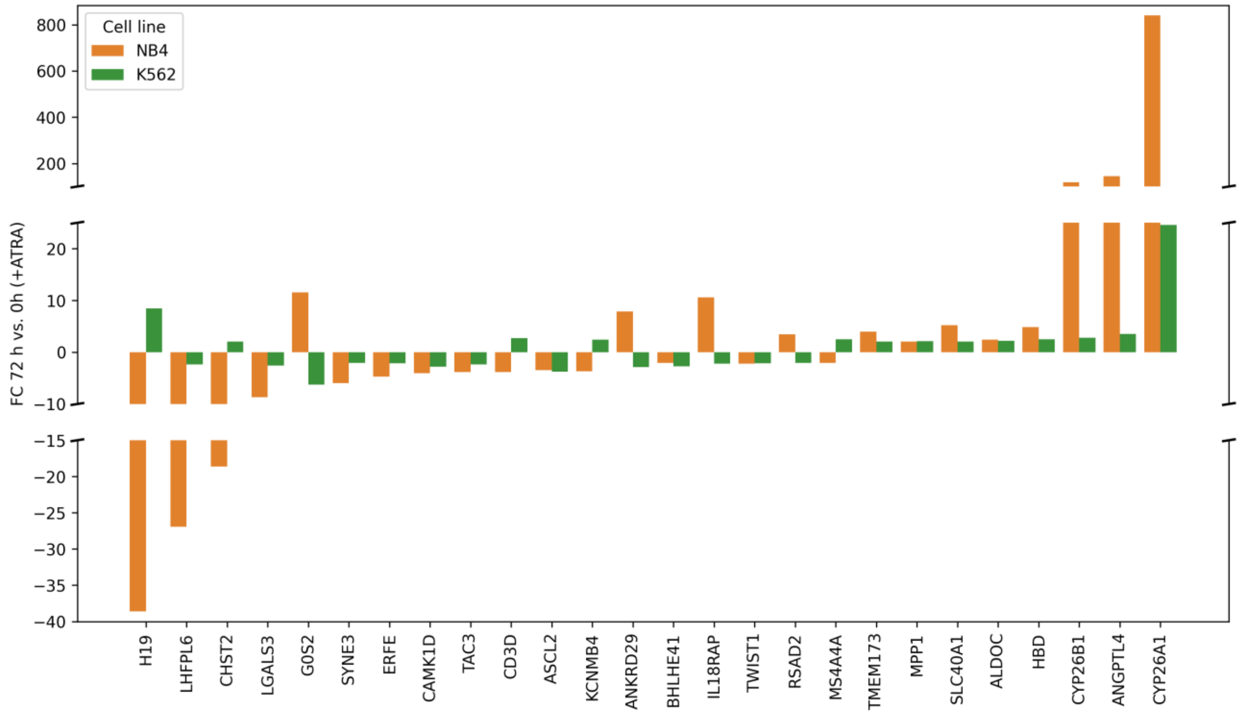

Figure S7. The transcriptomic profile of 26 differentially expressed genes (DEGs) that are common for ATRA-non-responsive cell line K562 and ATRA-responsive NB4 cell line at the 72 h after the ATRA treatment compared to control (0 h). FC - fold change (adjusted p-value <0.05).

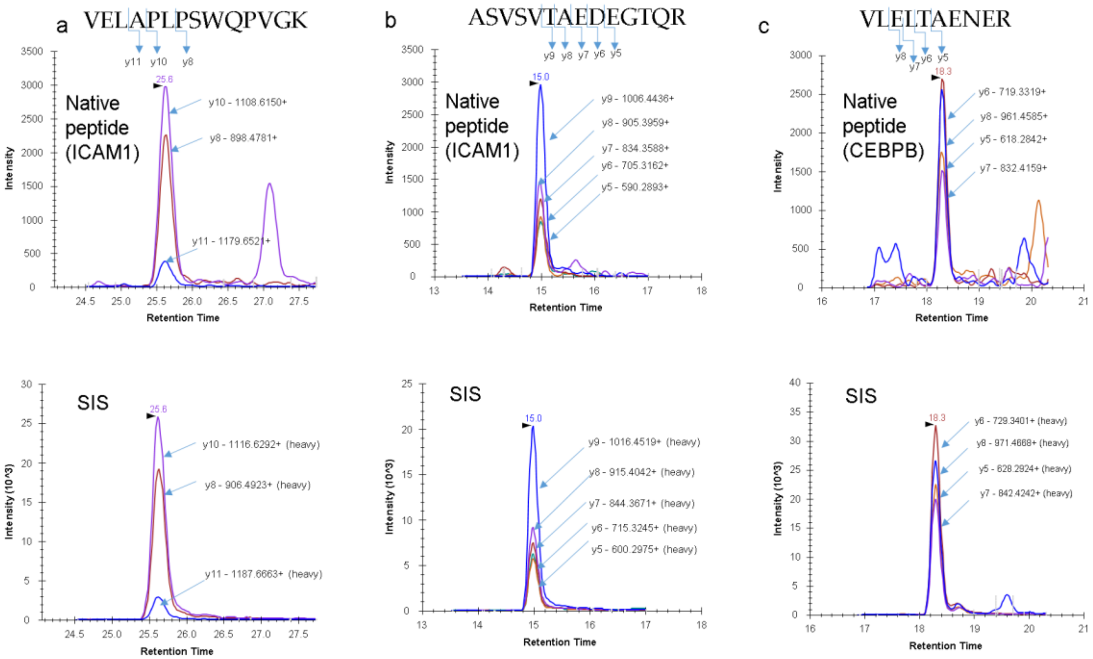

Figure S8. Trace of SRM transitions for native (upper panel) and stable isotope-labeled peptide standards (SISs) (lower panel) detected in cell lysate: (a, b) unique proteotypic peptides VELAPLPSWQPVGK and ASVSVTAEDEGTQR mapped to the ICAM1 protein; (c) unique proteotypic peptide VLELTAENER mapped to the CEBPB protein.

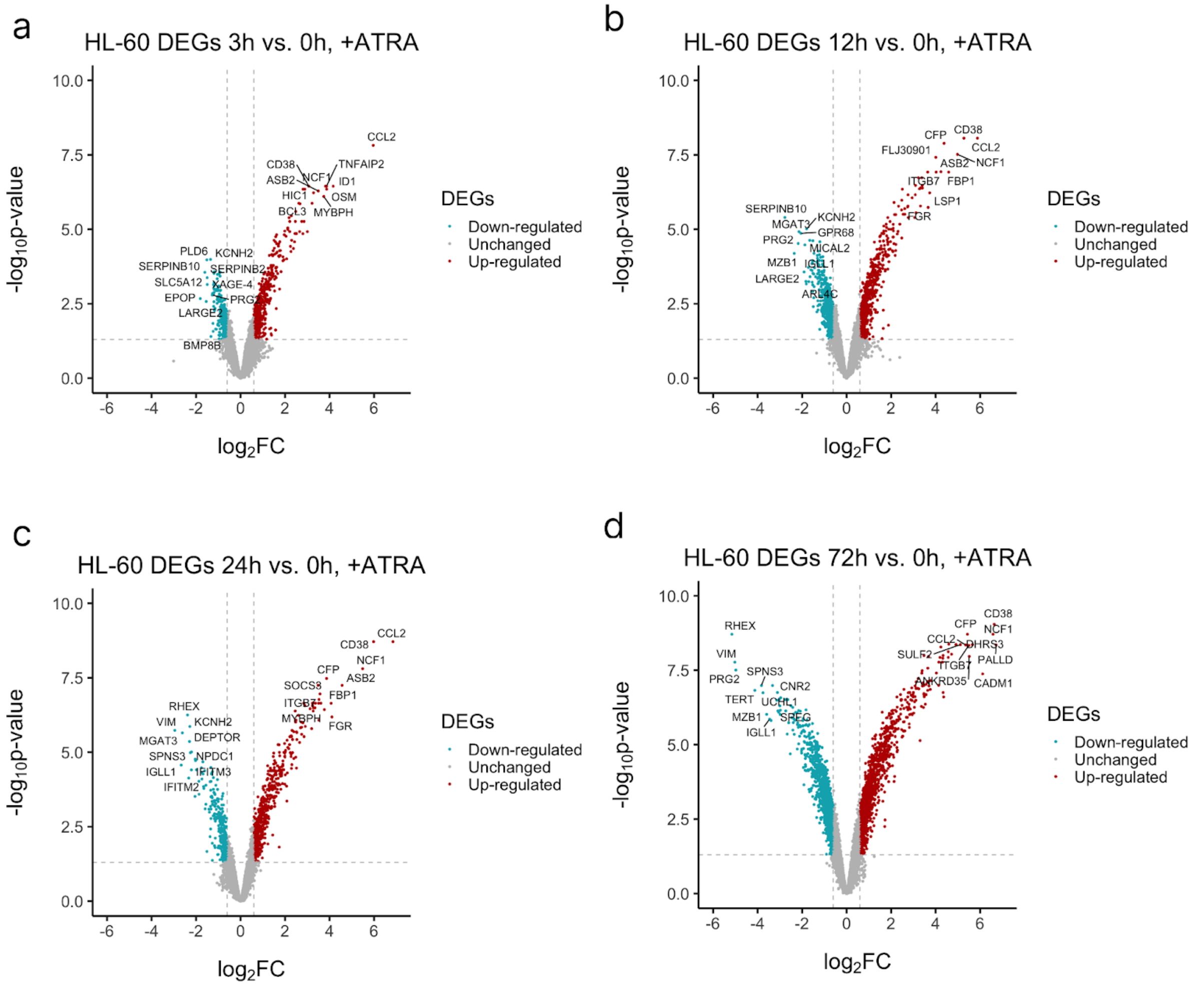

Figure S9. Volcano plots demonstrate differentially expressed genes (DEGs) of HL-60 cells at 3 h (a), 12 h (b), 24 h (c) , and 72 h (c) after the ATRA treatment. The names of top 10 down- and up-regulated DEGs(a) are shown.

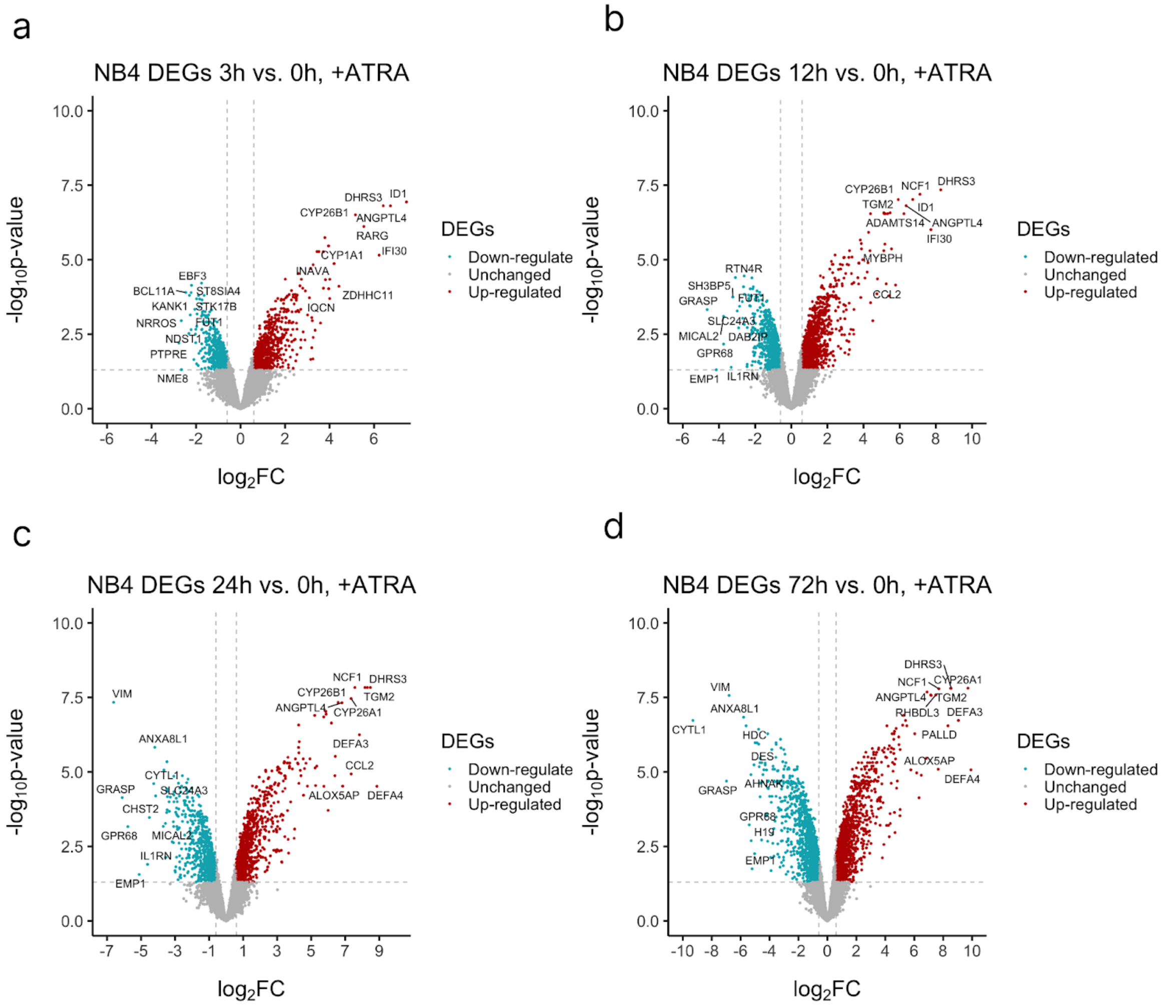

Figure S10. Volcano plots demonstrate differentially expressed genes (DEGs) of NB4 cells at 3 h (a), 12 h (b), 24 h (c) , and 72 h (c) after the ATRA treatment. The names of top 10 down- and up-regulated DEGs(a) are shown.

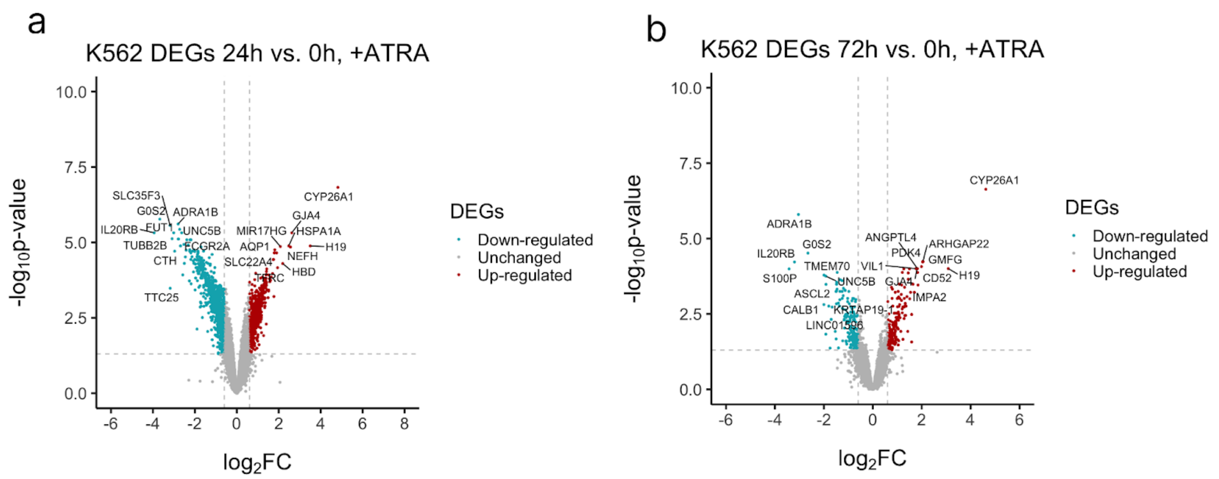

Figure S11. Volcano plots demonstrate differentially expressed genes (DEGs) of K562 cells at 24 h (a), and 72 h (b) after the ATRA treatment. The names of top 10 down- and up-regulated DEGs(a) are shown.

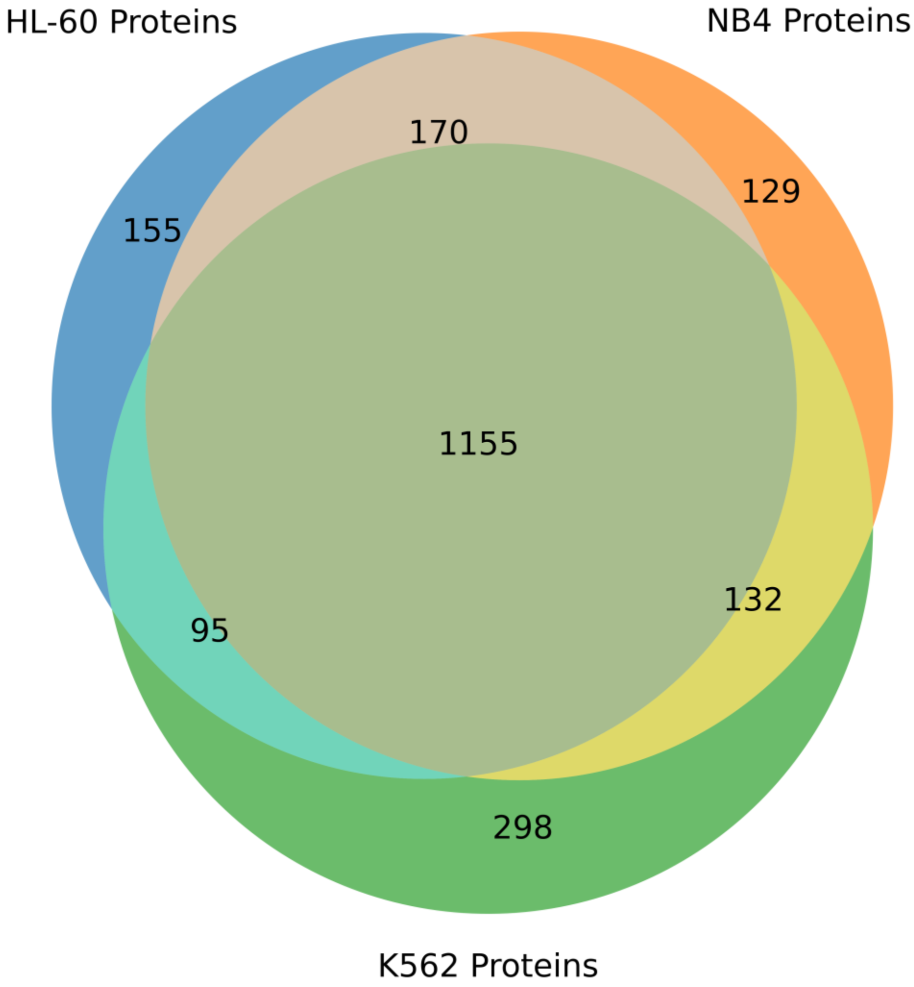

Figure S12. Venn diagram showing intersection of proteins that were identified in HL-60 (N = 1576), NB4 (N = 1590), and K562 (N = 1682) cells at least by two unique proteotypic peptides (FDR <0.01).

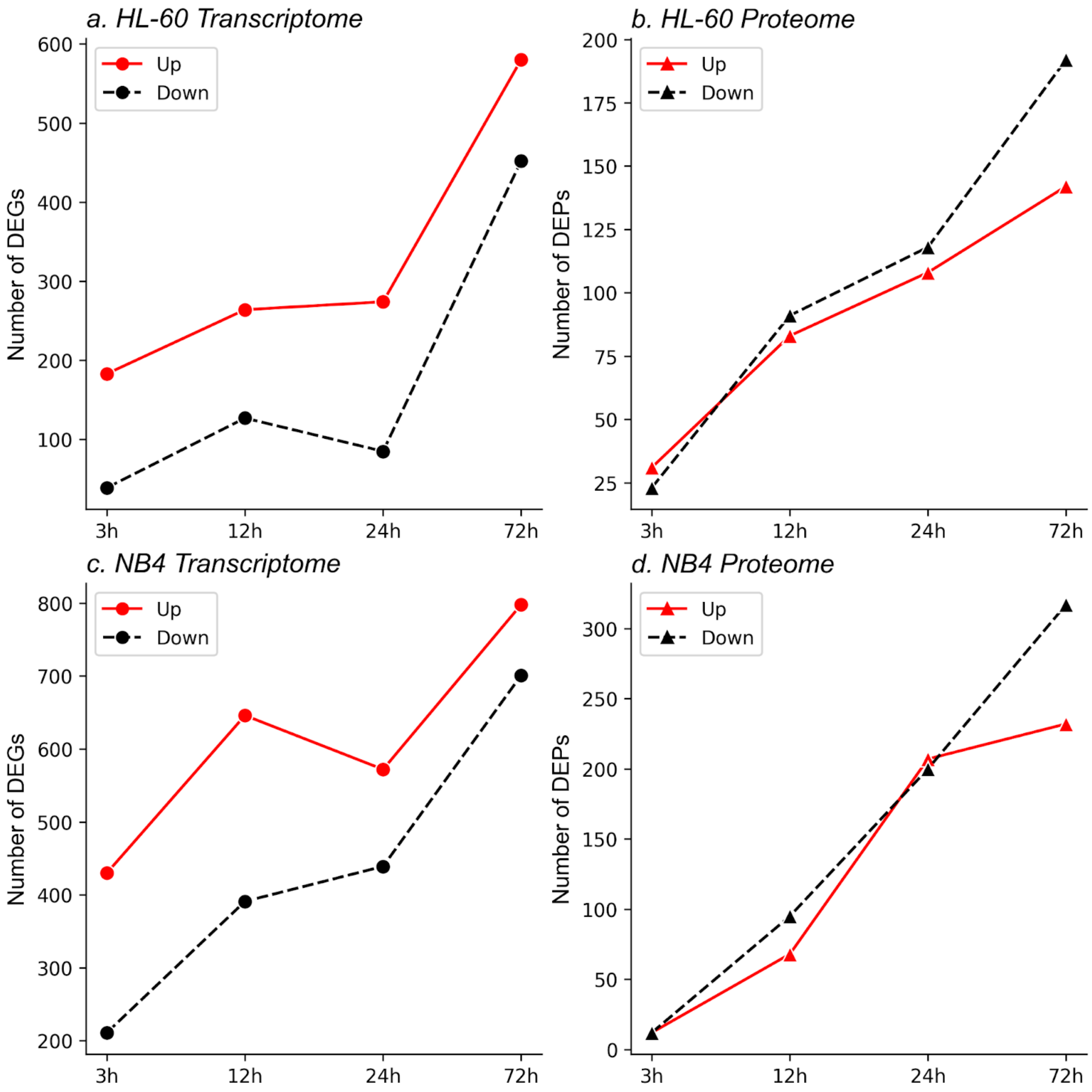

Figure S13. Number of differentially expressed genes (DEGs) and differentially expressed proteins (DEPs) detected in HL-60 and NB4 cell lines, which are responsive to the inducer of granulocytic differentiation, at 3, 12, 24 and 72 hours after the ATRA treatment.
